# CFTR Modulators Restore Acidification of Autophago-Lysosomes and Bacterial Clearance in Cystic Fibrosis Macrophages

**DOI:** 10.3389/fcimb.2022.819554

**Published:** 2022-02-16

**Authors:** Asmaa Badr, Mostafa Eltobgy, Kathrin Krause, Kaitlin Hamilton, Shady Estfanous, Kylene P. Daily, Arwa Abu Khweek, Ahmad Hegazi, Midhun N. K. Anne, Cierra Carafice, Frank Robledo-Avila, Youssra Saqr, Xiaoli Zhang, Tracey L. Bonfield, Mikhail A. Gavrilin, Santiago Partida-Sanchez, Stephanie Seveau, Estelle Cormet-Boyaka, Amal O. Amer

**Affiliations:** ^1^ Department of Microbial Infection and Immunity, College of Medicine, The Ohio State University, Columbus, OH, United States; ^2^ Clinical Pathology Department, College of Medicine, Mansoura University, Mansoura, Egypt; ^3^ Max Planck Unit for the Science of Pathogens, Berlin, Germany; ^4^ Biochemistry and Molecular Biology Department, Faculty of Pharmacy, Helwan University, Cairo, Egypt; ^5^ Department of Biology and Biochemistry, Birzeit University, West Bank, Palestine; ^6^ Center for Microbial Pathogenesis, Nationwide Children’s Hospital, Columbus, OH, United States; ^7^ Center for Biostatistics, Ohio State University, Columbus, OH, United States; ^8^ Department of Genetics and Genome Sciences, School of Medicine, Case Western Reserve University, Cleveland, OH, United States; ^9^ Department of Genetics and Genome Sciences, School of Medicine, Case Western Reserve University, Columbus, OH, United States; ^10^ Department of Veterinary Biosciences, College of Veterinary Medicine, The Ohio State University, Columbus, OH, United States

**Keywords:** autophagy, autophagosomes, lysosomal acidification, *Burkholderia cenocepacia* clearance, cystic fibrosis, CFTR modulators, autophago-lysosomes, macrophages

## Abstract

Cystic fibrosis (CF) human and mouse macrophages are defective in their ability to clear bacteria such as *Burkholderia cenocepacia*. The autophagy process in CF (F508del) macrophages is halted, and the underlying mechanism remains unclear. Furthermore, the role of CFTR in maintaining the acidification of endosomal and lysosomal compartments in CF cells has been a subject of debate. Using 3D reconstruction of z-stack confocal images, we show that CFTR is recruited to LC3-labeled autophagosomes harboring *B. cenocepacia.* Using several complementary approaches, we report that CF macrophages display defective lysosomal acidification and degradative function for cargos destined to autophagosomes, whereas non-autophagosomal cargos are effectively degraded within acidic compartments. Notably, treatment of CF macrophages with CFTR modulators (tezacaftor/ivacaftor) improved the autophagy flux, lysosomal acidification and function, and bacterial clearance. In addition, CFTR modulators improved CFTR function as demonstrated by patch-clamp. In conclusion, CFTR regulates the acidification of a specific subset of lysosomes that specifically fuse with autophagosomes. Therefore, our study describes a new biological location and function for CFTR in autophago-lysosomes and clarifies the long-standing discrepancies in the field.

**Graphical abstract:**
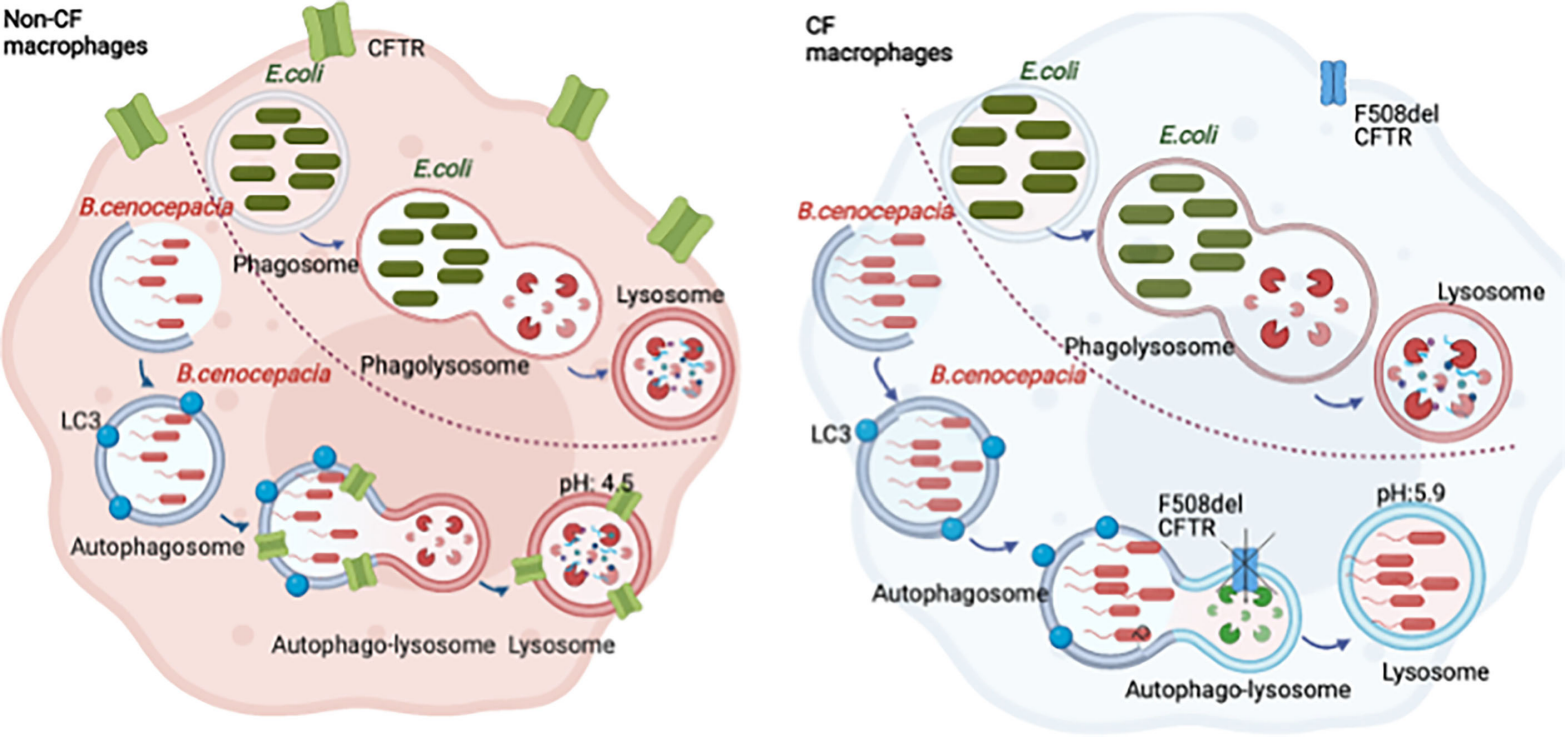
*Burkholderia cenocepacia* are enclosed in autophagosomes that fuse with the lysosomes forming autophago-lysosomes. The acidification of the autophago-lysosomes is dependent on CFTR function. *Escherichia coli* are enclosed in phagosomes that fuse with lysosomes, forming acidic phagosome-lysosomes independent of CFTR.

## Introduction

Cystic fibrosis (CF) is one of the most common lethal autosomal recessive diseases (Paranjape and Mogayzel, 2014; Byrne et al., 2015). It affects ≈80,000 people worldwide with nearly 1,000 newly diagnosed cases every year (Cystic Fibrosis Foundation, 2015). Chronic lung infections (Bruscia and Bonfield, 2016) and inflammation are hallmarks of CF patients (Bruscia et al., 2009; Bruscia and Bonfield, 2016; Castellani and Assael, 2017; Lévêque et al., 2017). The Cystic Fibrosis Transmembrane Conductance Regulator (CFTR) is an anion channel, located on the apical surface of epithelial cells and is expressed in human monocyte-derived macrophages (Del Porto et al., 2011), mouse alveolar macrophages, and bone-marrow-derived macrophages (BMDMs) (Bruscia et al., 2011). F508del mutation is the most common mutation affecting CF patients and is caused by the deletion of phenylalanine amino acid in position 508 (Castellani and Assael, 2017). F508del CFTR is expressed as a misfolded protein that becomes aggregated, malfunctional, and prematurely degraded (Lukacs and Verkman, 2012). CF patients carrying the F508del mutation experience infection with different respiratory pathogens including *Pseudomonas aeruginosa* (Hart and Winstanley, 2002; Del Porto et al., 2011; Bonfield et al., 2012), *Staphylococcus aureus* (Hart and Winstanley, 2002; Li et al., 2017), and *Burkholderia cenocepacia* (Scoffone et al., 2017). In healthy individuals, these pathogens can be cleared by robust autophagy activity within healthy immune cells (Abdulrahman et al., 2011) but can cause fatal infections in CF patients.

*B. cenocepacia* is a Gram-negative bacterium that affects approximately 3%–5% of CF patients (Scoffone et al., 2017). It can cause serious exacerbations called cepacia syndrome and is associated with a rapid decline in lung function (Ganesan and Sajjan, 2011). *B. cenocepacia* after being phagocytosed by healthy macrophages resides in bacteria-containing autophagosomes, which slowly fuse with lysosomes (Lamothe and Valvano, 2008). These vacuoles are characterized by multi-lamellar membranes and are labeled with autophagy markers such as Atg8/LC3 (Abdulrahman et al., 2011; Gatica et al, 2018). LC3 is a microtubule-associated protein that becomes recruited to the maturing phagophore, enhancing its maturation into an autophagosome (Klionsky et al., 2016; Yoshii and Mizushima, 2017). Autophagy is a homeostatic, essential, and conserved process by which cells can provide energy during stress (Klionsky et al., 2016; Harris et al., 2017). Intracellular molecules being degraded *via* autophagy include aggregated proteins, damaged organelles, and specific intracellular pathogens (Tooze and Yoshimori, 2010; Lamark and Johansen, 2012; Vural and Kehrl, 2014). This process involves sequestration of intra-cytoplasmic materials inside a double membrane structure called autophagosome (Klionsky et al., 2016; Yu et al., 2018). Autophagy is highly regulated by a group of autophagy-related (ATG) proteins that assemble into functional complexes, which are activated and recruited to phagophore membranes to initiate autophagy (Glick et al., 2010; Luciani et al., 2011). With autophagy stimulation, LC3 becomes lipidated and converted into LC3II, which is a marker for mature autophagosome (Mizushima and Yoshimori, 2007; Luciani et al., 2012). The autophagosome typically fuses with lysosomes to form an autophago-lysosome where the content is degraded. *B. cenocepacia* delays the fusion with lysosomes but eventually is degraded within specific vacuoles (autophago-lysosomes) in healthy macrophages (Lamothe and Valvano, 2008). In contrast, CF macrophages fail to clear *B. cenocepacia* largely due to inefficient autophagy (Abdulrahman et al., 2011; Abdulrahman et al., 2013; Tazi et al., 2016; Caution et al., 2019). The role of ion channels, including CFTR in sustaining proton flow to maintain the acidic pH of lysosomes, is unclear (Di et al., 2006; Steinberg et al., 2010). Additionally, the relation between deficient autophagy and CFTR mutation in CF macrophages is also unknown.

Using 3D reconstruction imaging, we demonstrated the localization of CFTR on the autophagosomes and autophago-lysosomes. We report here that CF macrophages have impaired lysosomal degradative capacity of *B. cenocepacia*, which resides in LC3-labeled autophagosomes but not of *Escherichia coli*, which are enclosed in vacuoles that do not acquire LC3. Interestingly, in healthy macrophages, *B. cenocepacia* vacuoles acquire CFTR. We demonstrate that CFTR is involved in the process of autophago-lysosomal acidification in macrophages. The absence of CFTR, in *cftr^−/−^* macrophages, or the presence of its F508del mutant in macrophages caused pH levels to be less acidic than in normal wild-type (WT) (CFTR^+/+^) macrophages. A combination of CFTR modulators that include a corrector, tezacaftor/VX-661 (Teza), which repairs misfolded CFTR protein, plus a potentiator, ivacaftor/VX-770 (Iva), which improves CFTR opening and increases chloride transport, is recently used in CF clinics (Clancy et al., 2019). Symdeko was approved in 2018 to treat CF patients who have at least one F508del mutation and is a combination of the CFTR corrector Teza and the potentiator Iva (Taylor-Cousar et al., 2017). Teza and Iva combination therapy is efficacious in improving pulmonary function in CF patients homozygous for the CFTR F508del mutation (Taylor-Cousar et al., 2017). We found that CFTR modulators enhance CFTR colocalization with RFP *B.c.* and restore lysosomal pH in F508del macrophages. This improvement in lysosomal acidification is reflected in improving autophagic flux. Furthermore, CFTR modulator treatment restored ion channel function at the plasma membranes and enhanced *B. cenocepacia* clearance in CF macrophages.

These results demonstrate previously unrecognized location and function for CFTR in specific lysosomes destined to fuse with autophagosomes and in autophago-lysosomes and demonstrate the effect of CFTR modulators on fundamental macrophage immune functions.

## Materials and Methods

### Bacterial Strains

*B. cenocepacia* K56-2 is a clinical isolate from a CF patient (Gislason et al., 2017). MH1K is a gentamicin-sensitive strain that is derived from K56-2. *B. cenocepacia* was a kind gift from Dr. Miguel Valvano at Queen’s University, Belfast (Hamad et al., 2010), and it was used in immunofluorescence colony-forming unit (CFU) experiments. K56-2 used in immunofluorescence experiments are complemented with a plasmid for red fluorescent protein (Ds-Red). Non-pathogenic *E. coli* BL21 and m-Cherry DH5α were used in immunofluorescence and CFU experiments. Bacterial cultures from all strains were grown overnight in Luria–Bertani (LB) media at 37°C and 200 rpm as previously described (Abdulrahman et al., 2011; Abdulrahman et al., 2013; Krause et al., 2018a).

### Human Monocyte-Derived Macrophages

Macrophages were derived from human blood monocytes as we previously described (Gavrilin et al., 2009). Cells were allowed to differentiate into monocyte-derived macrophages (MDMs) for 5 days at 37°C. MDMs were cultured in RPMI+ 10% AB serum, for 24 h, treated with CFTR modulators for 24 h, and then infected with *B. cenocepacia* either MH1K or K56 strains at a multiplicity of infection (MOI) of 10:1.

### Mice and Bone Marrow-Derived Macrophages

All experiments using animals were performed according to approved protocols from the Animal Care and Use Committee (IACUC) of The Ohio State University College of Medicine. WT C57BL/6 mice were obtained from Jackson Laboratories (Bar Harbor, MD, USA). F508del homozygous and *cftr^−/−^* S489X mice on a C57BL/6 background were obtained from Case Western Reserve University (Snouwaert et al., 1992; Zeiher et al., 1995; Hodges et al., 2008). *ATG5* ^flox/flox^-Lyz-*Cre* mice were a kind gift from Dr. Noburu Mizushima through Dr. Herbert W. Virgin from Washington University (Hara et al., 2006). The aforementioned mice were housed in the OSU vivarium. BMDMs were isolated as previously described (Abdulrahman et al., 2011; Abdulrahman et al., 2013; Krause et al., 2018a).

### Treatment and Infection of Primary Macrophages With CFTR Modulators

Primary mouse macrophages (BMDMs) were cultured in Iscove’s (IMDM media + 10% fetal bovine serum (FBS)). Cells were treated with CFTR modulators for 24 h at the following concentrations: 10 µM of tezacaftor (VX-661) (S7059, Selleckchem, Houston, TX, USA) and 5 µM of ivacaftor (VX-770) (S1144, Selleckchem). *In vitro* infections were performed as previously described (Krause et al., 2019; Estfanous et al., 2021).

### *In Vivo* Infection

Intratracheal infection was performed in *atg5^−/−^* mice anesthetized with isoflurane and inoculated with 100 µl of phosphate-buffered saline (PBS; Thermo Fisher Scientific, Waltham, MA, USA; 14,190,144) containing 10 × 10^6^ K56-2 *B. cenocepacia*. Bacterial load in organs was determined as follows: mice were sacrificed at 4 and 48 h post-infection to collect their lungs, livers, and spleens; homogenization in PBS was done as previously described (Abdulrahman et al., 2013; Krause et al., 2018a).

### Confocal Microscopy

Macrophages were fixed with 4% paraformaldehyde for 30 min. Cells were treated with 0.1% Triton X-100 for 20 min for permeabilization, followed by blocking with 5% goat serum in PBS. LC3A/B (4108, Cell Signaling Technology, Danvers, MA, USA), LAMP-1{1D4B} (ab25245, Abcam, Cambridge, UK), and CFTR CF3 (ab2784, Abcam) were visualized using goat IgG secondary antibody conjugated to Alexa Fluor 488, 594, 647 (A-11008, A-11007 Molecular Probes, Eugene, OR, USA). LysoTracker™ green (L7526, Molecular Probes) was used to stain acidic compartments of infected macrophages. Nuclei were stained with 1 µg/ml of 4′,6′-diamino-2-phenylindole (DAPI; D1306, Molecular Probes). Images were captured using a laser scanning confocal fluorescence microscope with a ×60 objective (Olympus Fluoview FV10i) as previously described (Krause et al., 2018a).

### CFTR Localization Imaging

Fluorescent images were captured on Olympus FV 3000 inverted microscope using 60×/1.4 NA oil objective. Images were taken at z-sections of 0.5- to 1-μm intervals by using the 488-nm CFTR (ab2784, Abcam), RFP *B. cenocepacia*, LAMP-1{1D4B} (ab25245, Abcam), LC3 (4108, CST), and 405-nm (DAPI) lasers. Image reconstructions of z-stacks were generated in Imaris software (Bitplane, Inc., Belfast, UK). CFTR colocalization with *B. cenocepacia*, LC3, and LAMP-1 was analyzed using the colocalization tool of Imaris as previously described (Asaithamby et al., 2011). Briefly, z-sections were first assembled, and then the threshold for each channel was calculated by using the Surpass function. Subsequently, the threshold values were used to build a colocalization channel, and the percent of volume colocalized between the two channels was extracted from the colocalization channel statistics.

### Immunoblotting

Macrophages were lysed in TRIzol reagent, and proteins were separated according to the manufacturer’s instructions as previously described (42). Membranes were probed for LC3 (4108S, Cell Signaling Technology), LAMP-1{1D4B} (ab25245, Abcam), ATP6V1B2 {D307Q} (1448, Cell Signaling Technology), and GAPDH (14C10, Cell Signaling Technology). Protein bands were detected with secondary antibodies conjugated to horseradish peroxidase, followed by enhanced chemiluminescence reagents (RPN2209, Amersham, Piscataway, NJ, USA). Densitometry analyses were performed by normalizing target protein bands to their respective loading control (GAPDH) using ImageJ software as previously described (Tazi et al., 2016; Krause et al., 2018a).

### Lysosomal Acidification Measurement

*LysoSensor experiments*: Macrophages were seeded in a 96-well Costar, black, clear-bottom plate at a cell density of 100,000/well, and cells were treated for 24 h with CFTR modulators. Bafilomycin-A1 (Baf-A1; Enzo Life Sciences, Farmingdale, NY, USA; BML-CM110-0100) and rapamycin (Sigma Aldrich, St. Louis, MO, USA; R0395) were administered at 100 nM and 5 µg/ml concentration for 2 and 1 h, respectively. LysoSensor Green DND-189 (L7535, Molecular Probes) was incubated with macrophages in imaging solution (10 mM of HEPES, 1 mg/ml of bovine serum albumin (BSA), 1 mg/ml of glucose, 1 mM of MgCl_2_, and 1.8 mM of CaCl_2_ in PBS) for 10 min at a concentration of 1 µM. Macrophages were then washed 2× with PBS and incubated in imaging solution for 15–30 min. LysoSensor fluorescence was measured using SpectraMaxi3x micro-plate reader (Molecular Devices, San Jose, CA, USA) at 443/505 and then normalized to cell number counted using a SpectraMax MiniMax 300 Imaging Cytometer.

### pH Calibration Curve

Macrophages were incubated with LysoSensor Green DND-189 as before, and then calibration buffers with different pH values were added to the assigned wells for 10 min before reading the plate as before. The composition of buffers used for generating the pH calibration curve was as follows: 125 mM of KCl, 25 mM of NaCl, 10 µM of monensin, 10 μM of nigericin, and 25 mM of *N*-[2-hydroxyethyl]-piperazine-*N*-[2-ethanesulfonic acid] (HEPES; pH 7.5 or 7.0), or 25 mM of 2-[*N*-morpholino] ethanesulfonic acid (MES; pH 6.5, 6.0, 5.5, 5.0, 4.5, 4.0, or 3.5). Each buffer solution is adjusted to the appropriate final pH using 1 N of NaOH or 1 N of HCl.

### Lysosomal Proteolytic Activity

Macrophages were seeded in 96-well (3603, Corning, Corning, NY, USA), black, clear-bottom plates loaded with 10 µg/ml of DQ Green-BSA (D-12050, Molecular Probes) for 16 h in full media, followed by washing 2× with PBS. Cells were incubated in serum-free media for 2 h of chase period. The fluorescence intensity of the dye was measured at 505/530 using a plate reader and normalized to cell count as described before. Confocal microscopy imaging was done using the fluorescein isothiocyanate (FITC) channel. Intensities of DQ-BSA were measured using ImageJ software. Graphs depict Integrated Density (Int. Den.), which is defined as the “product of area and Mean Gray Value” of the pixel values in the selected area. Int. Den. of DQ-BSA was normalized to cell number.

### Cathepsin B Activity Assay

Macrophages were seeded in 6-well plates, treated with CFTR modulators for 24 h, and then lysed using lysis buffer. Protein lysate measuring 10 µg was used, and the manufacturer’s protocol was followed to measure cathepsin B activity (ab65300, Abcam).

### Cathepsin D Activity Assay

Macrophages were seeded in a 96-well, black, clear-bottom, plate at a density of 100,000 per well. Bodipy FL-pepstatin A (P12271, Thermo Fisher Scientific) (1 µM) in imaging solution was incubated with cells for 10 min. Fluorescence intensity was measured at 500/525 nm using a plate reader, and then the readings were normalized to cell count as described before.

### Lactate Dehydrogenase Cytotoxicity Assay

Lactate dehydrogenase (LDH) released from infected macrophages with MH1K *B. cenocepacia* was measured using CytoTox-ONE Homogeneous Membrane Integrity Assay (G7891, Promega, Madison, WI, USA) according to the manufacturer’s protocols. Calculation of *B. cenocepacia*-induced LDH release was done as follows; % LDH = ((infected sample − respective negative control)/(respective positive control − respective negative control)) * 100.

### Transmission Electron Microscopy

Macrophages were seeded in Permanox Lab-Tek chamber slides (177,429, Nunc) and then fixed with 2.5% glutaraldehyde (18,426, Ted Pella, Redding, CA, USA) in 0.1 M of phosphate buffer, pH 7.4 (S369 and S37, Fisher Scientific) containing 0.1 M of sucrose (S2-500, Fisher Scientific). Campus Microscopy & Imaging Facility at The Ohio State University performed sample processing as previously described (Krause et al., 2018a). FEI Tecnai G2 Spirit transmission electron microscope plus AMT camera system was used for taking images.

### Whole-Cell Patch-Clamp Recording

Whole-cell patch-clamp experiments were performed on WT and F508CFTR mouse BMDMs. The cells were treated with 10 μM of tezacaftor (VX-661) and 5 μM of ivacaftor (VX-770) for 24 h. The currents were recorded with a patch amplifier (Multiclamp 700B). Step protocol consisted of 400-ms voltage from −80 to +80 mV from a holding potential of −40 mV. The pipettes were pulled from a borosilicate glass capillary tubing (TW150-3, World Precision Instruments, Sarasota, FL, USA) using puller P-97 (Sutter Medical Technologies, Atlanta, GA, USA), and the pipettes had a resistance of 3–5 MΩ. The intrapipette solution contained 139 mM of CsCl, 2 mM of MgCl, 5 mM of EGTA, 10 mM of HEPES, 5 mM of glucose, 2 mM of ATP, and 1 mM of GTP at pH 7.2. The bath solution was prepared with 145 mM of NaCl, 15 mM of sodium glutamate, 4.5 mM of KCl, 1 mM of MgCl, 2 mM of CaCl_2_, 10 mM of HEPES, and 5 mM of glucose at a pH of 7.4. For the activation of CFTR current, a cocktail solution was prepared with 1 μM of forskolin, 10 μM of cAMP, 100 μM of IBMX, and 2 mM of ATP. The analysis was performed by using Clampfit 11.0.3 software.

### Statistics

Data were analyzed using GraphPad Prism 8.0. Figures display SEM from at least three independent experiments. Comparisons between groups were conducted with Student’s t-test or one-way/two-way ANOVA. *p*-Values <0.05 were considered statistically significant.

## Results

### CFTR Localizes to LC3-Labeled Autophagosomes and *Burkholderia cenocepacia*-Containing Autophagosomes in Human Macrophages

**Figure 1:**
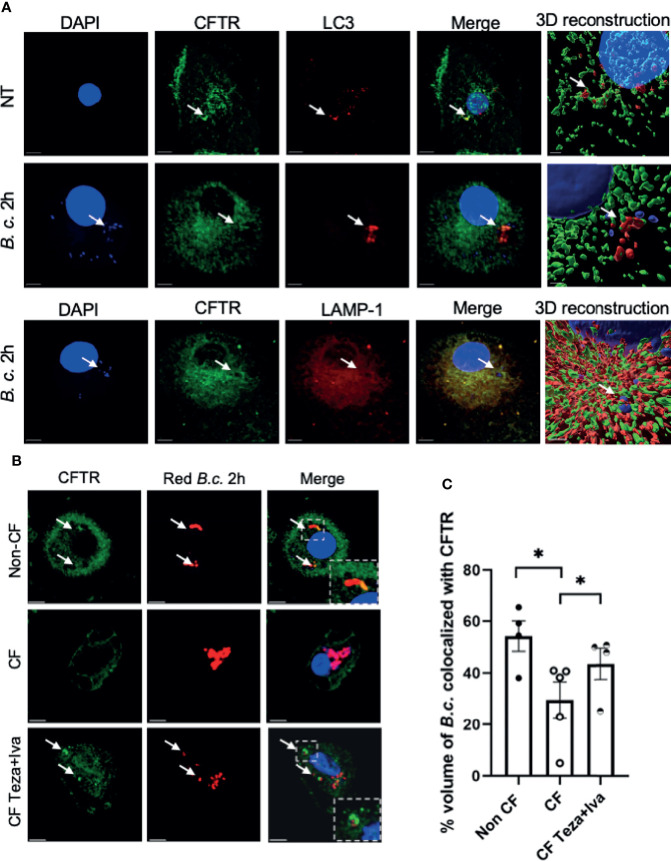
CFTR localizes to LC3-labeled autophagosomes and *Burkholderia cenocepacia*-containing autophagosomes and autophago-lysosomes. **(A)** Representative projection and 3D reconstruction from z-stack confocal microscopy images of human non-cystic fibrosis (non-CF) macrophages, either non-infected (NT) or infected with *B. cenocepacia* (MH1K) for 2 h (*B. c.* 2 *h*). Macrophages were fixed and stained for CFTR (Alexa Fluor-488), LC3, and LAMP-1 (Alexa Fluor-594); DAPI was used to stain *B. c.* DNA. White arrows point to CFTR colocalized with LC3, *B. c.*, or LAMP-1 (n = 3 biological replicates). Scale bar = 5 µm. **(B)** Representative confocal microscopy images of human non-CF, CF NT, and CF treated with Teza+Iva for 24 h macrophages. Macrophages were infected with *B. cenocepacia* expressing RFP for 2 h (red *B. c*. 2h) and stained for CFTR. White arrows point to CFTR colocalized with red *B. c.* **(C)** % volume of red *B. c.* colocalized with CFTR measured from images in panel **(B)** Data represent mean ± SEM calculated from 3D reconstructed images using Imaris software from at least 5 randomly chosen fields of view with an average of 30 cells per field (n = 4 non-CF, n = 5 CF NT, and n = 4 CF Teza+Iva). Statistical analysis was performed using a linear mixed-effects model (REML); *, *p* ≤ 0.05.

Previous studies have shown that CFTR is expressed in macrophages under resting conditions (Bajmoczi et al., 2009). Phagocytosis of latex beads caused CFTR to accumulate at the site of bead entry (Di et al., 2006). Furthermore, CFTR colocalized with *P. aeruginosa* during internalization into epithelial cells (Kowalski and Pier, 2004). To detect if CFTR is recruited to vacuoles that express autophagy markers in human macrophages, we used confocal microscopy and stained CFTR and the autophagy marker LC3. We used a CFTR antibody that was validated for its specificity in knock-out cells and was used by several publications for CFTR immunofluorescence staining (Zhang et al., 2018; Borcherding et al., 2019). We found that CFTR colocalized with LC3-labeled puncta (autophagosomes) in non-infected non-CF macrophages (**Figure 1A**). Additionally, non-CF macrophages were infected with *B. cenocepacia* and stained for CFTR, autophagosomal and lysosomal markers LC3 and LAMP-1, respectively. CFTR colocalized with autophagosomes and lysosomes that contained *B. cenocepacia*, as shown in **Figure 1A** with bacterial DNA stained with DAPI (indicated by arrows). The percent volume of LC3 colocalized with CFTR in non-infected cells had a mean of 48.6 and a standard error of the mean (SEM) of 5.46, and it increased significantly after 2 h of *B. cenocepacia* infection to reach a mean of 72.5 and a SEM of 7.68 (**Supplemental Figure 1E**). Additionally, LAMP-1 percentage volume colocalized with CFTR had a mean of 68 and a SEM of 6.078. Furthermore, we infected human macrophages with Ds-Red Fluorescent Protein (RFP) expressing *B. cenocepacia*. We found that the percentage volume of *B. cenocepacia* colocalized with CFTR was significantly more in non-CF than in CF macrophages (**Figures 1B, C**). Additionally, treatment of CF macrophages with CFTR modulators (Teza+Iva) prior to their infection with *B. cenocepacia* increased the percentage volume of *B. cenocepacia* colocalized with CFTR, measured by Imaris from z-stack images (**Figures 1B, C**). Together, our data demonstrate that *B. cenocepacia* resides in autophagosomes that acquire CFTR.

### CFTR Is Essential for Maintaining Autophago-Lysosomal Acidity in Macrophages and Responds to CFTR Modulators

**Figure 2:**
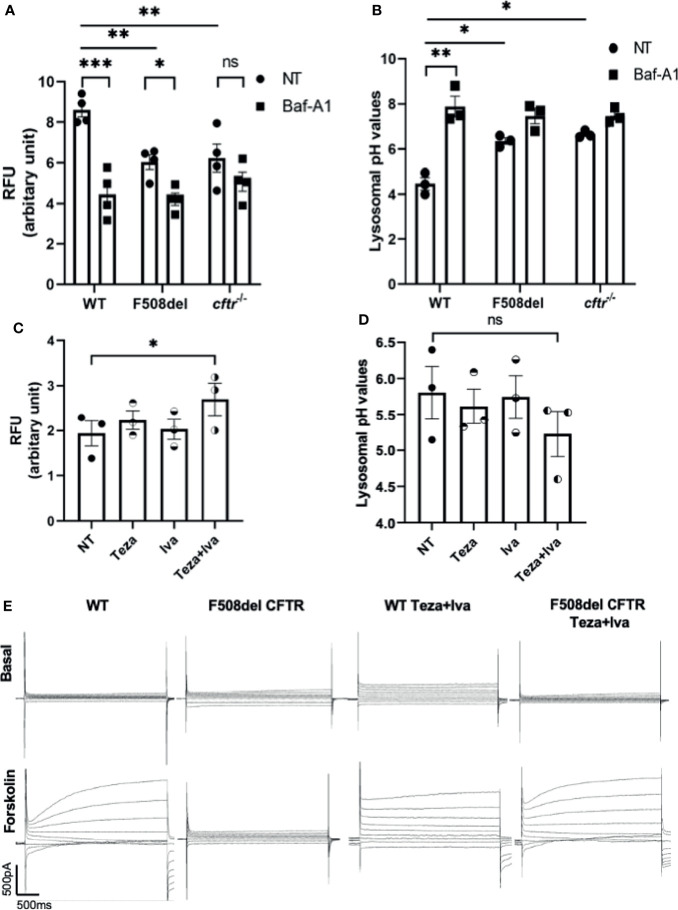
CFTR is essential for maintaining autophago-lysosomal acidity in macrophages and responds to CFTR modulators. **(A)** Mean fluorescence intensity (MFI) of LysoSensor Green DND-189 in wild-type (WT), F508del, and *cftr^−/−^* macrophages either non-infected (NT) or treated with Baf-A1. Data show MFI normalized to the number of cells. Data represent mean ± SEM (n = 4 biological replicates). Statistical analysis was performed using two-way ANOVA; *, *p* ≤ 0.05; **, *p* ≤ 0.01; ***, *p* ≤ 0.001; ns, non-significant. **(B)** Corresponding lysosomal pH values in WT, F508del, and *cftr^−/−^* macrophages stained with LysoSensor Green DND-189. Data represent MFI normalized to number of cells and plotted to the pH calibration curve. Data represent mean ± SEM (n = 3 biological replicates). Statistical analysis was performed using two-way ANOVA; *, *p* ≤ 0.05; **, *p* ≤ 0.01. **(C)** MFI of LysoSensor Green DND-189 in F508del macrophages treated with 10 µM of Teza, 5 µM of Iva, or both for 24 h. Data represent mean ± SEM (n = 3 biological replicates). Statistical analysis was done using one-way ANOVA; *, *p* ≤ 0.05. **(D)** Corresponding lysosomal pH values in F508del macrophages NT or treated with the indicated CFTR modulators for 24 h. Data represent mean ± SEM (n = 3 biological replicates). Statistical analysis was performed using one-way ANOVA; ns, non-significant. **(E)** WT or F508del CFTR macrophages were seeded in coverslips, and F508del macrophages were treated with 10 μM of Teza and 5 μM of Iva for 24 h. Representative current traces for CFTR chloride channel conductance in WT and F508del macrophages are shown as basal state and after the addition of a cocktail (shown as forskolin) containing 1 μM of forskolin, 10 μM of cAMP, 100 μM of IBMX, and 2 mM of ATP. Step protocol consisted of 400-ms voltage from −80 to +80 mV from a holding potential of −40 mV. The traces show the basal and activated CFTR current in WT, F508del CFTR, WT Teza+Iva, and F508del CFTR Teza+ Iva macrophages (n = 3 biological replicates).

The localization of CFTR on autophagosomes and autophago-lysosomes suggests that it contributes to their acidification. It was proposed that autophagy in F508del macrophages is impaired due to the sequestration of autophagy molecules in CFTR aggregates (Abdulrahman et al., 2013). However, the autophagy defect in CF macrophages may be due to compromised lysosomal function owing to defective acidification of autophago-lysosomes when CFTR is mutated. To test the role of CFTR in maintaining lysosomal acidification, LysoSensor Green DND-189, a specific lysosomal probe was used. This probe accumulates specifically in acidic organelles, and its fluorescence intensity increases with acidity. First, we tested the lysosomal acidification in F508del and CFTR knock-out (*cftr^−/−^*) murine macrophages in comparison to their WT counterparts. The fluorescence intensity of LysoSensor Green was significantly less in F508del and *cftr^−/−^* than in WT macrophages (**Figure 2A**). Treatment with Baf-A1, the vacuolar ATPase inhibitor (Mauvezin and Neufeld, 2015), significantly decreased lysosomal acidification in WT and F508del macrophages (**Figure 2A**). We then measured the actual pH values in lysosomes using a pH calibration curve. Our data demonstrate that lysosomal pH levels in WT macrophages had an average of ~4.5, whereas in F508del and *cftr^−/−^* macrophages, the average lysosomal pH values were ~5.9, and ~6.5, respectively, and Baf-A1 increased lysosomal pH values to ~7.0 (**Figure 2B**). We then treated F508del macrophages with CFTR modulators to test the efficacy of these drugs in improving lysosomal acidification. Interestingly, the acidification was improved when using a combination of tezacaftor + ivacaftor (Teza+Iva) (**Figure 2C**). Accordingly, lysosomal pH values were more acidic, ~5.1, in Teza+Iva-treated F508del macrophages than in non-treated (NT) ones (**Figure 2D**). Together, these results demonstrate that F508del macrophages have defective lysosomal acidification that improves in response to CFTR modulators; however, pH improvement did not reach the WT level with CFTR modulators treatment. Moreover, we assessed the lysosomal acidity in the presence of *B. cenocepacia* infection in WT, F508del NT, and modulator-treated macrophages at 4 h of infection using the lysosomal marker LysoSensor Green. We found that the acidity in F508del macrophages was significantly less than that in WT macrophages during *B. cenocepacia* infection. Additionally, treatment with Teza+Iva improved the acidity in F508del lysosomes as evidenced by the increased fluorescence of the lysosomal marker (**Supplemental Figure 1A**). Albeit, when we incubated the cells with *E. coli* conjugated with pHrodo Green to measure the acidity inside the vacuoles contained *E. coli* in WT, F508del, and *cftr^−/−^* macrophages, there was no difference in the fluorescence intensity of pHrodo *E. coli* between NT WT, F508del, and *cftr^−/−^* macrophages. Treatment with Baf-A1 caused a significant reduction in fluorescence since it inhibits vacuolar ATPase and alkalinizes all subsets of lysosomes (**Supplemental Figure 1B**). However, treatment with the autophagy enhancer, rapamycin did not affect the fluorescence intensity. These data confirm that CFTR acidifies *B. cenocepacia* but not *E. coli*-containing vacuoles.

### CFTR Modulators Improve CFTR Cl^−^ Conductance in Mouse Macrophages

The effect of CFTR modulators on improving the function of mouse F508del CFTR channel conductance was tested using “gold standard” patch-clamp techniques. We performed whole-cell patch-clamp experiments on WT and F508del macrophages that were either NT or treated with Teza+Iva. We found that forskolin stimulated cAMP-dependent conductance of chloride in WT macrophages (**Figure 2E**). In contrast, F508del macrophages failed to respond to forskolin stimulation. However, Teza+Iva treatment of F508del macrophages restored the ability of these cells to produce chloride currents, similar to the conductance of WT macrophages in response to forskolin (**Figure 2E**). These data confirm that mouse F508del CFTR channels are in fact responsive to the effect of CFTR modulators and that the combination of a CFTR corrector and a potentiator restores the CFTR-mediated Cl^−^ conductance in F508del macrophages.

### Lysosomal Degradative Function in F508del Macrophages Is Defective and Improves in Response to CFTR Modulators

**Figure 3:**
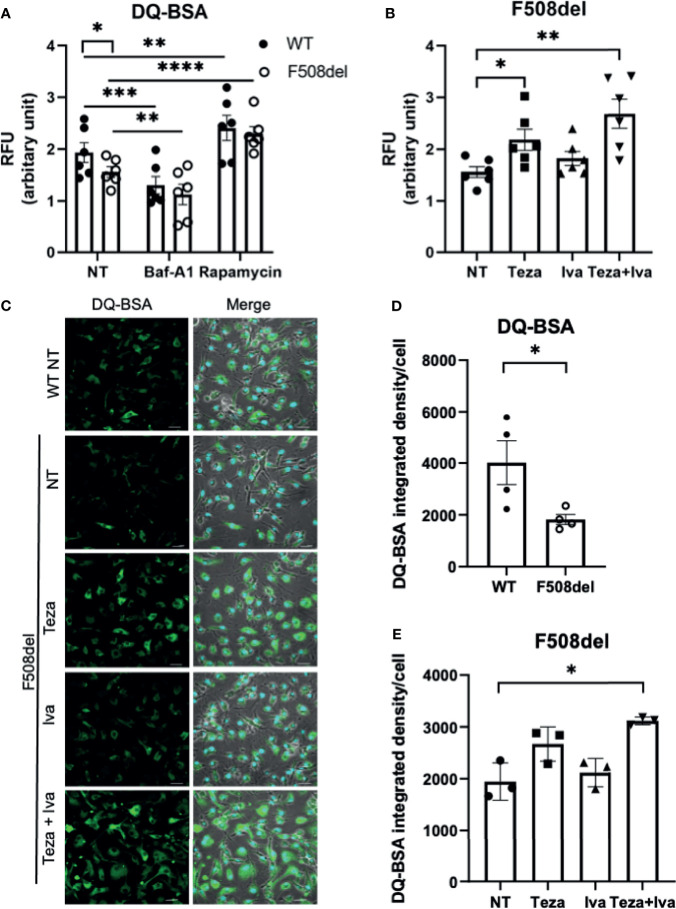
Lysosomal degradative function in F508del macrophages is defective and improves in response to CFTR modulators. **(A)** DQ-BSA fluorescence in wild-type (WT) and F508del macrophages, non-infected (NT), treated with 100 nM of Baf-A1 for 2 h, or with 5 µg/ml of rapamycin for 24 h. Data show mean fluorescence intensity (MFI) normalized to the total number of cells. Data represent mean ± SEM (n = 6 biological replicates). Statistical analysis was using two-way ANOVA; *, *p* ≤ 0.05; **, *p* ≤ 0.01; ***, *p* ≤ 0.001; ****, *p* ≤ 0.0001. **(B)** DQ-BSA fluorescence in F508del macrophages NT (plotted in A or treated with 10 µM of Teza −/+ 5 µM of Iva for 24 h. Data represent MFI normalized to the total number of cells. Data represent mean ± SEM (n = 6 biological replicates). Statistical analysis was done using one-way ANOVA; *, *p* ≤ 0.05; **, *p* ≤ 0.01. **(C)** Representative confocal microscopy images of DQ-BSA staining of WT, and F508del macrophages, NT, or treated with the indicated CFTR modulators (n = 3 biological replicates); scale bar = 20 µm. Merged images showing phase contrast, DAPI, and DQ-BSA channels. **(D, E)** DQ-BSA fluorescence integrated density calculated from images in **(C)** normalized to the total number of cells. Data represent mean ± SEM calculated using ImageJ software from randomly chosen fields of view with an average of 50 cells per field from 3 independent experiments. Statistical analysis was done using paired t-test **(D)** and one-way ANOVA **(E)**; *, *p* ≤ 0.05.

A crucial function of macrophages is the degradation of intracellular cargo (Vural and Kehrl, 2014). The degradative and recycling function of autophagy in macrophages depends upon the trafficking of intracellular vacuoles and their fusion with the degradative compartments, including lysosomes (Frost et al., 2017). Since F508del macrophages exhibit ineffective autophagy (Abdulrahman et al., 2011; Abdulrahman et al., 2013), along with defective autophago-lysosomal acidification, the assessment of the lysosomal and autophagic degradative ability in these macrophages is important. To accomplish this, we employed DQ-Green-BSA (DQ-BSA), which is composed of a BSA derivative conjugated to a fluorophore, which self-quenches and its fluorescence increases upon digestion of BSA. This allows autophagy-mediated proteolysis to be monitored through capturing brightly fluorescent BSA fragments (Frost et al., 2017). DQ-BSA fluorescence was measured using a microplate spectro-fluorometer as well as confocal microscopy. We used Baf-A1 as a negative control since it inhibits autophagy and rapamycin as a positive control for autophagy stimulation. The spectro-fluorometer results show less DQ-BSA fluorescence intensity in F508del compared to WT macrophages (**Figure 3A**). Treatment with Baf-A1 caused a significant decrease in DQ-BSA fluorescence, whereas rapamycin enhanced the fluorescence intensity in both WT and F508del macrophages (**Figure 3A**). We also noticed a marked increase in the fluorescence intensity of DQ-BSA in Teza+Iva-treated F508del macrophages (**Figure 3B**). To account for possible differences in BSA uptake between WT and F508del macrophages that can affect fluorescence levels in addition to proteolysis, we used another BSA conjugate, BSA AF-647. This conjugate is labeled with a stable fluorophore that is insensitive to proteolysis. Therefore, the emitted fluorescence should reflect the total amounts of BSA uptake. We found no significant differences in the uptake of BSA-647 between WT and F508del macrophages whether treated or not with CFTR modulators (**Supplemental Figure 1C**). Confocal microscopy images showed similar results of impaired DQ-BSA proteolysis in CF macrophages that was improved with CFTR modulators treatment (**Figure 3C**). WT cells had a significantly more fluorescence integrated density of DQ-BSA than F508del macrophages, and their treatment with Teza+Iva significantly enhanced the fluorescence intensity (**Figures 3D, E**). Therefore, these data show that lysosomal proteolytic degradative function is defective in F508del macrophages and can be improved by CFTR modulators. Overall, these results confirm the role of CFTR in lysosomal ability to degrade delivered cargo.

### The Expression of Lysosomal Proteins Is Similar Between Wild Type and F508del Macrophages

Defective acidification in F508del macrophages can be attributed to defective lysosomal biogenesis and/or impaired expression of vacuolar ATPase (V-ATPase) in lysosomes. To discern between these possibilities, we performed immunoblot analysis for the late endosomal and lysosomal markers LAMP-1 (Appelqvist et al., 2013) as well as for V-ATPase that transports protons to the inside of lysosomes for maintaining high luminal acidity (Kissing et al., 2018). We treated WT and F508del macrophages with Baf-A1 to detect the effect of lysosomal acidification impairment on the expression level of these proteins. Densitometry analysis of immunoblots showed no significant differences in the expression levels of LAMP-1 between WT and F508del macrophages whether NT or treated with Baf-A1 (**Supplemental Figures 2A, B**). Furthermore, we found that Baf-A1 treatment in WT macrophages significantly increased vacuolar ATPase (B2 subunit) expression level, but not in F508del macrophages. While the V-ATPase level was slightly increased in F508del macrophages, CFTR modulators treatment showed no significant effect on its expression level (**Supplemental Figures 2C, D**). These data support our conclusion that CFTR contributes to the acidification of lysosomes and that defective lysosomal acidification in F508del macrophages is not due to lower lysosomal mass or decreased V-ATPase expression levels.

### The Lysosomal Enzyme Cathepsin D Shows Reduced Proteolytic Activity in F508del Macrophages

**Figure 4:**
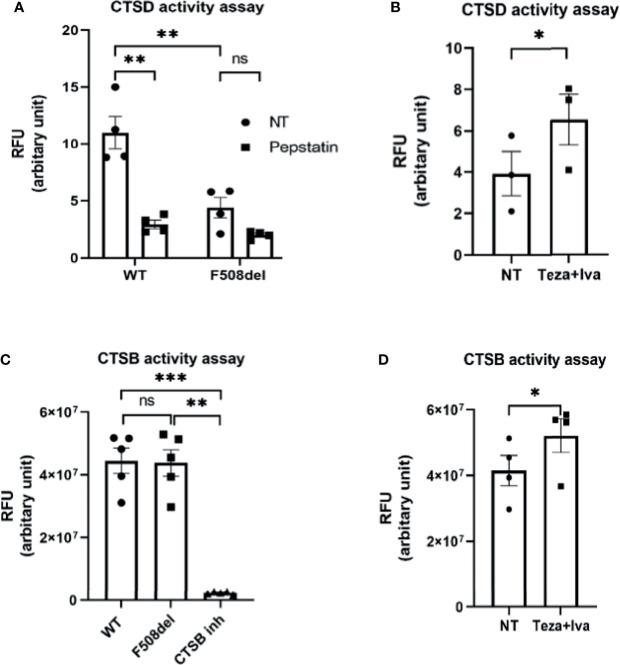
The lysosomal enzyme cathepsin D shows reduced proteolytic activity in F508del macrophages. **(A)** Cathepsin D (CTSD) activity in wild-type (WT) and F508del macrophages either non-infected (NT) or treated with pepstatin (20 µg/ml). Data show mean fluorescence intensity (MFI) of Bodipy-pepstatin normalized to the number of cells. Data represent mean ± SEM (n = 4 biological replicates). Statistical analysis was done using two-way ANOVA; **, *p* ≤ 0.01; ns, non-significant. **(B)** CTSD activity in F508del macrophages NT (plotted in **A**) or treated with Teza + Iva. Data show MFI of Bodipy-pepstatin normalized to the number of cells. Data represent mean ± SEM (n = 3 biological replicates). Statistical analysis was done using one-way ANOVA; *, *p* ≤ 0.0.5. **(C)** Cathepsin B (CTSB) activity in WT and F508del either NT or treated with CTSB inhibitor. Data show mean fluorescence intensity per 10 µg of total protein. Data represent mean ± SEM (n = 5 biological replicates). Statistical analysis was performed using two-way ANOVA; **p ≤ 0.01; ***p ≤ 0.001; ns, non-significant. **(D)** CTSB activity in F508del either NT (plotted in **C**) or treated with Teza+Iva for 24 h. Data show MFI per 10 µg of total protein. Data represent mean ± SEM (n = 4 biological replicates). Statistical analysis was performed using paired t-test; *, *p* ≤ 0.05.

Cathepsins are lysosomal enzymes responsible for proteolytic degradation within lysosomes (Szulc-Dąbrowska et al., 2020). They have an essential role in autophagy, cellular stress signaling, and lysosomal-dependent cell death (Turk et al., 2012). Several members of the cathepsin family are found in lysosomes. Cathepsin D (CTSD) and cathepsin B (CTSB) are aspartic and cysteine proteases, respectively (Aghdassi et al., 2018). They are secreted as inactive zymogens, and in an optimal pH environment, their cleavage is promoted, leading to an increase in the active mature form (Turk et al., 2012; Aghdassi et al., 2018). To specifically measure CTSD activity, we used the fluorescent marker BODIPY-FL-pepstatin (Chen et al., 2000). Pepstatin is a selective marker for active CTSD, and its fluorescent label BODIPY TR-X-casein releases highly fluorescent BODIPY dye-labeled peptides upon enzymatic digestion. The activity of CTSD is proportional to the increase of its fluorescence (Chen et al., 2000). F508del and WT macrophages were either NT or treated with non-fluorescent pepstatin as a negative control to inhibit CTSD activity. Notably, the activity of CTSD was markedly decreased in F508del macrophages, and treatment with pepstatin inhibited CTSD activity (**Figure 4A**). Interestingly, treatment of F508el macrophages with Teza+Iva significantly improved CTSD activity (**Figure 4B**). Measuring the activity of CTSB, we found no difference in CSTB activity between WT and F508del macrophages (**Figure 4C**). Nevertheless, there was a significant increase in CTSB activity in F508del macrophages when they were treated with Teza+Iva (**Figure 4D**). We concluded that the activity of CTSD is impaired due to the defective acidity inside lysosomes of F508del macrophages. Additionally, CFTR modulators improved the lysosomal acidity and improved the activity of both CTSD and CTSB.

### Autophagy Activity in Macrophages Is Essential for Restricting *Burkholderia cenocepacia In Vivo* and *In Vitro*

**Figure 5:**
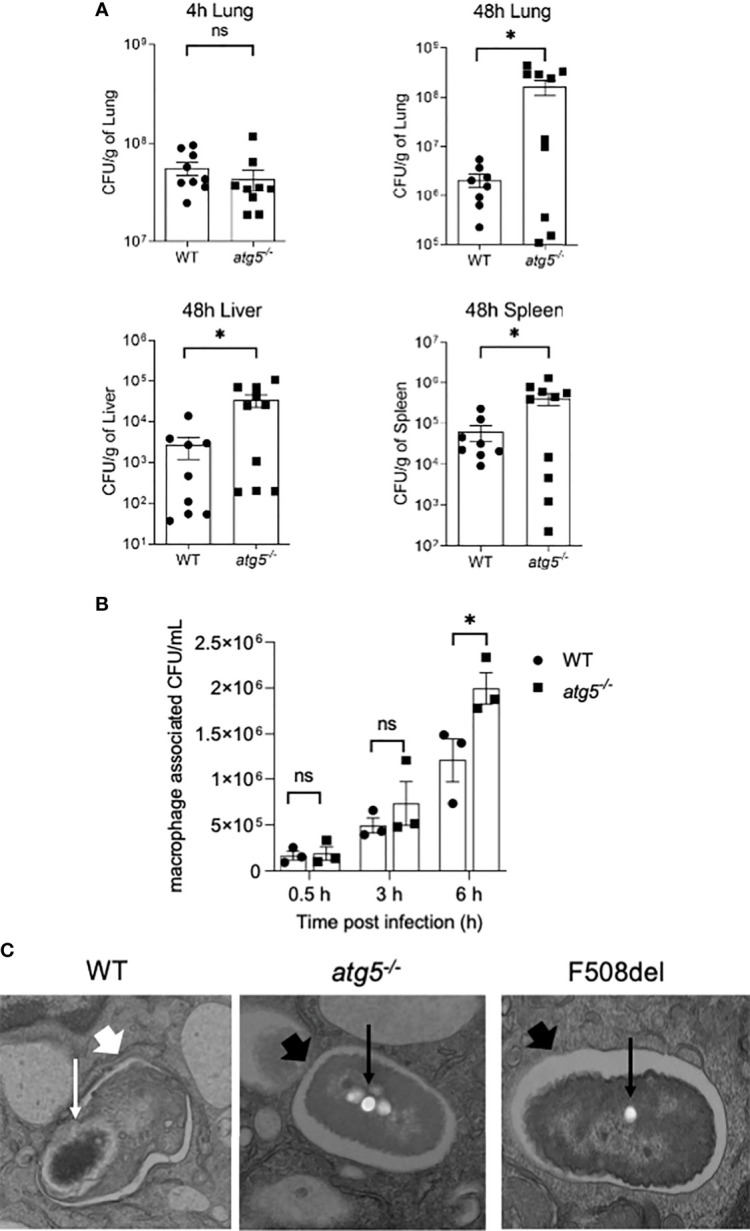
Autophagy activity in macrophages is essential for restricting *Burkholderia cenocepacia in vivo* and *in vitro*. **(A)** Colony-forming units (CFUs) in lung, liver, and spleen from wild-type (WT) and *atg5^−/−^* mice intratracheally infected with *B. c.* at indicated time points. Data represent mean ± SEM (n = 5 biological replicates). Statistical analysis was performed using a two-tailed Student’s t-test; *, *p* ≤ 0.05; ns, non-significant. **(B)** Intracellular survival of *B. cenocepacia* in WT and *atg5^−/−^* macrophages at 0.5, 3, and 6 h. Data represent mean ± SEM (n = 3 biological replicates). Statistical analysis was performed using two-way ANOVA; *, *p* ≤ 0.05; ns, non-significant. **(C)** Qualitative transmission electron microscopy images of *B. c*. infected WT, *atg5^−/−^*, and F508del macrophages at 2 h post-infection. White arrows indicate a multilamellar membrane characteristic for autophagosomes, and the small arrows indicate the degraded bacteria. Black arrow indicates a single-membrane vacuole with intact bacteria indicated by small black arrows.

CF macrophages elicit weak autophagic activity, which improves upon stimulation with rapamycin, resulting in improved *B. cenocepacia* clearance (Abdulrahman et al., 2011; Abdulrahman et al., 2013). To verify the role of autophagy in macrophages as an important host defense mechanism against *B. cenocepacia*, we used a myeloid cell-specific ATG5 conditional knockout, *ATG5* ^flox/flox^-Lyz2-*Cre* mice (*atg5^−/−^*). These mice lack ATG5 only in myeloid cell lineage, while all other cells express normal *Atg5* genes. We examined the role of efficient autophagy in myeloid cells in controlling *B. cenocepacia* replication and dissemination *in vivo*. We intratracheally infected *atg5^−/−^* mice with *B. cenocepacia* K-56 strain and then harvested the lung, liver, and spleen at different time points post-infection. Although similar numbers of *B. cenocepacia* CFUs were recovered at 4 h post-infection in *atg5^−/−^* mice lungs, which reflect an equal initial inoculum, these mice exhibited higher *B. cenocepacia* loads in their lungs at 48 h post-infection (**Figure 5A**). Furthermore, enhanced dissemination of *B. cenocepacia* to the liver and spleen was observed in a*tg5^−/−^* mice at 48 h post-infection (**Figure 5A**). Furthermore, we compared the intracellular survival of *B. cenocepacia* in *atg5^−/−^* BMDMs. **Figure 5B** shows that the uptake of *B. cenocepacia* at 0.5 h was comparable in both WT and *atg5^−/−^* macrophages. However, at 6 h post-infection the absence of ATG5 caused a significant increase in intracellular bacterial numbers (**Figure 5B**). *E. coli* clearance, on the other hand, was not impaired due to the absence of ATG5 in *atg5^−/−^* BMDMs. **Supplemental Figure 3A** shows that both WT and *atg5^−/−^* BMDMs were equally efficient in clearing *E. coli* at 6 h of infection. Therefore, a functional autophagy system in macrophages controls *B. cenocepacia* intracellular replication and dissemination *in vivo* and *in vitro*.

To confirm the identity of the *B. cenocepacia* vacuole, we imaged *B. cenocepacia*-infected macrophages using electron microscopy (EM). Macrophages from WT, *atg5^−/−^*, and F508del CFTR mice were infected with *B. cenocepacia* for 2 h and then processed for EM imaging. The representative images in **Figure 5C** show a multi-lamellar autophagosome enclosing compromised-looking bacteria in WT macrophages. However, in F508del macrophages, healthy-looking bacteria enclosed within single membrane vacuoles similar to those in *atg5^−/−^* macrophages were found. These data confirm that *B. cenocepacia* is enclosed in autophagosomes in WT but not in F508del or *atg5^−/−^* macrophages.

### CFTR Modulators Restore Defective Autophagy in F508del Macrophages

**Figure 6:**
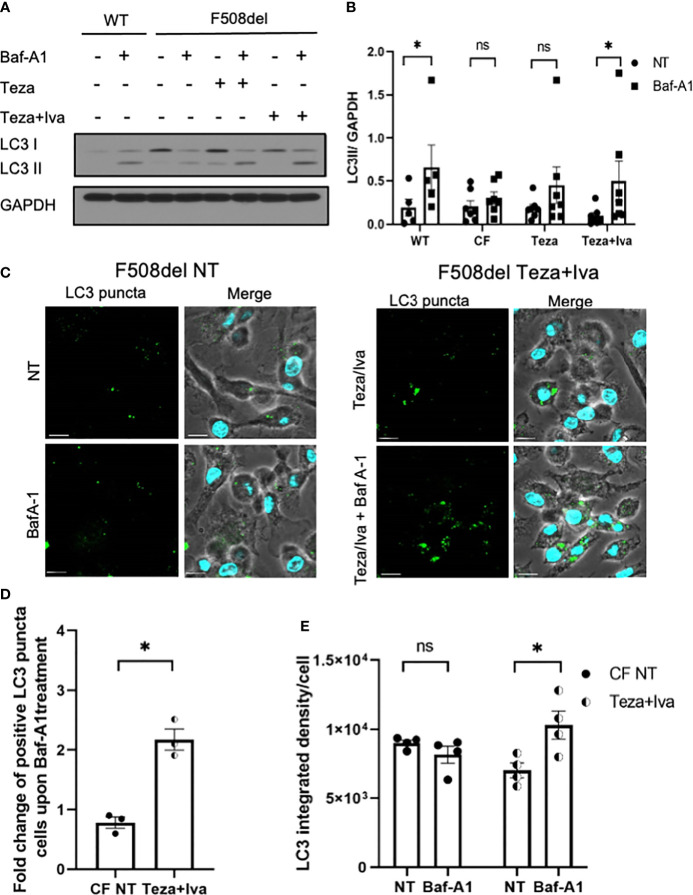
CFTR modulators restore defective autophagy in F508del macrophages. **(A)** Representative LC3 immunoblot from wild-type (WT) and F508del macrophages treated with either vehicle (non-infected (NT)) or 10 µM of Teza −/+ 5 µM of Iva for 24 h, followed by −/+100 nM of Baf-A1 (n = 4 biological replicates). **(B)** Densitometry analysis for LC3II in WT and F508del macrophages shown **(A)**, representing the ratio of LC3II to GAPDH. Data represent mean ± SEM (n = 5 WT, n = 7 cystic fibrosis (CF)). Statistical analysis was performed using two-way ANOVA; *, *p* ≤ 0.05; **, *p* ≤ 0.01; *ns, non-significant.* **(C)** Confocal microscopy images showing LC3 puncta accumulation in F508del macrophages either NT or treated with Teza+Iva −/+ Baf-A1 (100 nM) (n = 3 biological replicates). Scale bar = 10 µm. **(D)** Percentage of cells expressing >5 LC3 puncta in F508del macrophages shown in panel **(C)** Data represent mean ± SEM calculated by scoring at least 200 cells from randomly chosen fields of view, normalized to the total number of cells from 3 independent experiments. Statistical analysis was performed using paired t-test; *, *p* ≤ 0.05. **(E)** LC3 integrated density in F508del macrophages either NT or Teza+Iva treated −/+ Baf-A1. Data represent mean ± SEM calculated using ImageJ software from at least 5 randomly chosen fields of view with approximately 200 cells from 5 independent experiments. Statistical analysis was performed using one-way ANOVA; *, *p* ≤ 0.05; *ns, non-significant*.

Given that autophagy is defective in CF macrophages (Abdulrahman et al., 2011; Abdulrahman et al., 2013; Tazi et al., 2016) and epithelial cells (Luciani et al., 2010), we then determined if CFTR modulators would improve autophagy activity in F508del macrophages. To evaluate autophagic flux in response to CFTR modulators, F508del macrophages were treated with Teza−/+Iva for 24 h. Subsequently, these cells were either left NT or treated with Baf-A1 to prevent lysosomal fusion and block autophagic flux (Mizushima and Yoshimori, 2007; Klionsky et al., 2016). The flux was assessed by identifying the difference in LC3II accumulation before and after treatment with Baf-A1 (Klionsky et al., 2016). LC3 immunoblot analysis revealed that the autophagic flux in F508del macrophages was impaired in comparison to WT cells. LC3 II accumulation after treating with Baf-A1 increased in CFTR modulators (Teza+Iva)-treated F508del cells (**Figures 6A, B**). In addition, we analyzed LC3 puncta formation in response to (Teza+Iva) treatment in the presence of Baf-A1 in F508del macrophages by confocal microscopy (Krause et al., 2018a). We calculated the percentage of macrophages with >5 LC3 puncta (Abdulrahman et al., 2013; Tazi et al., 2016; Krause et al., 2018b). In accordance with immunoblot results, Teza+Iva treatment significantly increased the number of macrophages with positive LC3 puncta upon Baf-A1 treatment compared to NT F508del macrophages (**Figures 6C, D**). Moreover, Teza+Iva treatment of F508del macrophages lead to a significant increase in LC3 signal intensity measured as integrated density with the addition of Baf-A1 in comparison to the NT cells, indicating that the autophagic flux was restored (**Figure 6E**). Notably, the expression of the *Map1lc3b* gene was found to be similar between WT and F508del macrophages, and it was not significantly changed in response to Teza−/+Iva (**Supplemental Figure 1D**). This indicates that CFTR modulators do not increase the transcription of the autophagy marker LC3, and the autophagy stimulation by them is not transcriptionally regulated. Altogether, our data indicate that CFTR modulators restore impaired autophagy in F508del macrophages without increasing the expression of the autophagy-related gene LC3.

### CFTR Modulators Reduce *Burkholderia cenocepacia* Burden in CF Macrophages and Decrease Bacterial-Associated Cell Death

**Figure 7:**
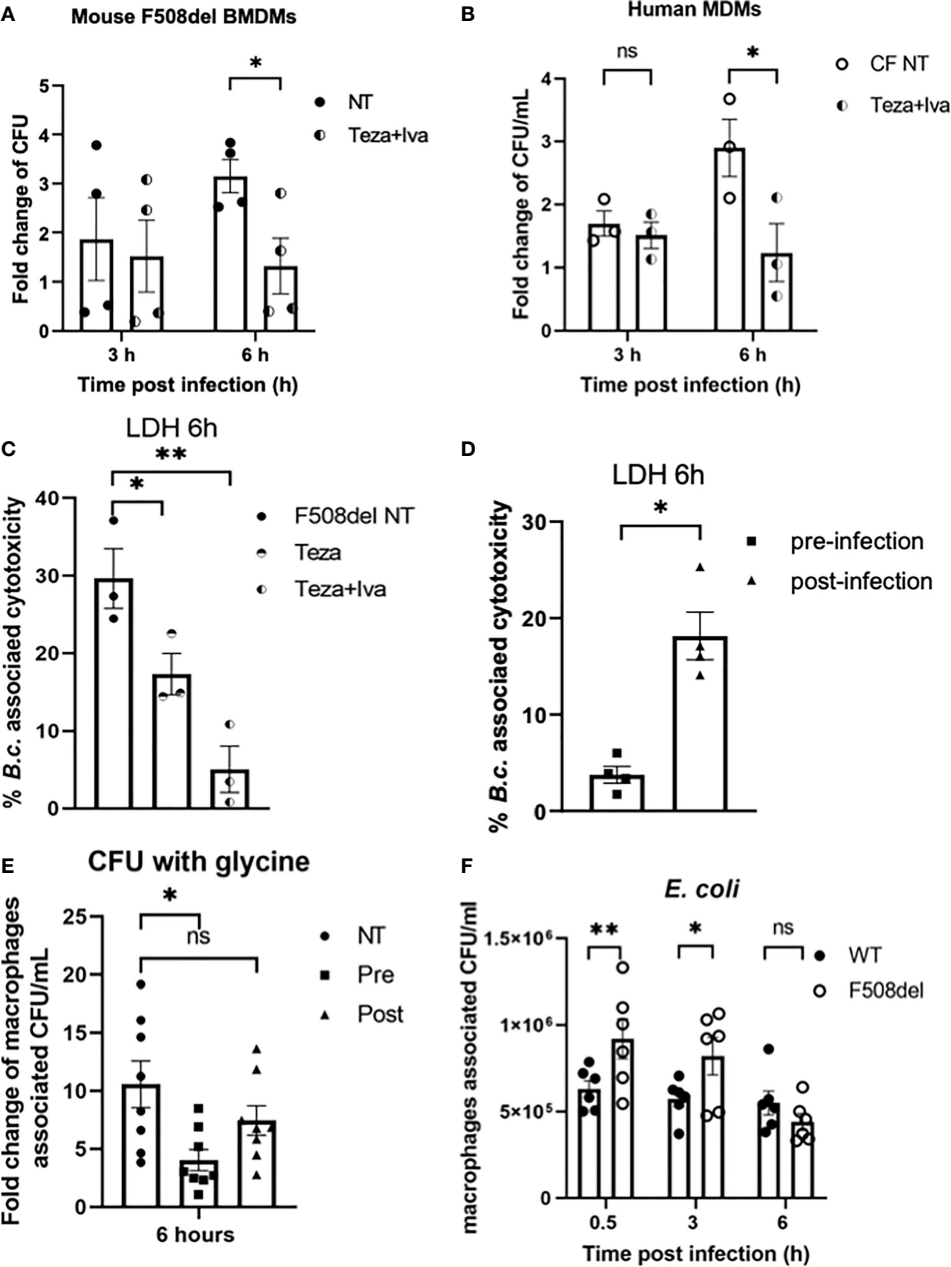
CFTR modulators reduce *Burkholderia cenocepacia* burden in cystic fibrosis (CF) macrophages and decrease bacteria-associated cell death. **(A)** Fold change of *B. c.* intracellular survival in mouse F508del macrophages treated with Teza+Iva for 24 h prior to infection for 3 and 6 h. Fold change was calculated relative to 0.5-h time point (invasion). Data represent mean ± SEM (n = 5 biological replicates). Statistical analysis was performed using two-way ANOVA; *, *p* ≤ 0.05. **(B)** Fold change of *B. c.* intracellular survival in human CF^F508del/F508del^ monocyte-derived macrophages (MDMs) either non-infected (NT) or treated with Teza+Iva for 24 h. Fold change was calculated relative to 0.5-h time point (invasion). Data represent mean ± SEM (n = 3 biological replicates). Statistical analysis was performed using two-way ANOVA; *, *p* ≤ 0.05; ns, non-significant. **(C)** Lactate dehydrogenase (LDH) release at 6 h from *B.c.*-infected mouse F508del macrophages either NT or treated with Teza−/+Iva for 24 h prior to their infection. Data represent mean ± SEM (n = 3 biological replicates). Statistical analysis was performed using two-way ANOVA; *, *p* ≤ 0.05; **, *p* ≤ 0.01. **(D)** LDH release at 6 h from *B.c.*-infected mouse F508del macrophages, treated with Teza+Iva either prior to (pre-infection) or after infection (post-infection). Data represent mean ± SEM (n = 4 biological replicates). Statistical analysis was performed using two-way ANOVA; *, *p* ≤ 0.05. **(E)** Fold change of intracellular survival of *B. c.* in mouse F508del macrophages at 6 h of infection that were NT, pre-, or post-infection treated with Teza+Iva. Fold change was calculated relative to 0.5-h time point (invasion). Data represent mean ± SEM (n = 4 biological replicates). Statistical analysis was performed using two-way ANOVA; *, *p* ≤ 0.05; ns, non-significant. **(F)** Intracellular survival of *Escherichia coli* in wild-type (WT) and F508del macrophages at 0.5, 3, and 6 h of infection. Data represent mean ± SEM (n = 3 biological replicates). Statistical analysis was done by two-way ANOVA; *p ≤ 0.05; **p ≤ 0.01; ns, non-significant.

Since *B. cenocepacia* is cleared *via* autophagy and our data show that CFTR modulators restore chloride channel conductance and improve autophagy in F508del macrophages, we examined their effect on bacterial clearance. Murine F508del macrophages were treated with Teza+Iva and then infected with *B. cenocepacia* (MH1K strain) in the presence of the drugs throughout the course of infection as described previously (Abdulrahman et al., 2011; Abdulrahman et al., 2013). In our previous publications (Abdulrahman et al., 2011; Abdulrahman et al., 2013; Tazi et al., 2016), we demonstrated that the bacterial loads of *B. cenocepacia* were higher in F508del macrophages compared to their WT counterparts. Here, we found that Teza+Iva treatment increases the number of *B. cenocepacia* that were phagocytosed by macrophages. At 0.5 h post-infection, Teza+Iva-treated F508del macrophages had significantly more intracellular *B. cenocepacia* CFUs (**Supplemental Figure 3B**). In addition, the presence of Teza+Iva significantly reduced *B. cenocepacia* intracellular CFUs at 6 h compared to the NT cells (**Figure 7A**). To test if CFTR modulators had a similar effect on bacterial clearance in human CF macrophages, we treated human CF^F508del/F508del^ MDMs with Teza+Iva for 24 h before infecting them with *B. cenocepacia*. We found that macrophage-associated CFUs were also markedly decreased in CFTR modulator-treated human CF macrophages when compared to their NT counterparts (**Figure 7B**). In addition, we tested the effect of CFTR modulators on the survival of murine F508del macrophages in response to *B. cenocepacia* infection. We found that these macrophages released less LDH at 6 h of infection when they were pretreated with Teza−/+Iva. This indicates that CFTR modulators reduce *B. cenocepacia*-induced cytotoxicity and cell death (**Figure 7C**). Overnight incubation of CFTR modulators with *B. cenocepacia* in LB media did not affect bacterial growth (**Supplemental Figure 3C**). Therefore, the improved bacterial clearance is mediated by macrophages and is not due to the direct bactericidal effects of CFTR modulators on *B. cenocepacia*.

Since in the experiments described above Teza+Iva treatments were performed before infection, we then tested if treating CF macrophages with CFTR modulators post-infection with *B. cenocepacia* would still promote bacterial clearance. In addition, we measured LDH release (cell death) associated with *B. cenocepacia* infection in F508del macrophages when they were treated with modulators prior (pre) and after (post) infection. We found that treatment with CFTR modulators post-*B. cenocepacia* infection was not as effective as the pretreatment in reducing macrophage death (**Figure 7D**). To exclude the effect of cell death on *B. cenocepacia* replication in CF macrophages, we treated CF macrophages with glycine 1 h before infection and kept it throughout the course of the experiment as described before (Estfanous et al., 2021). Glycine is a cytoprotective agent that prevents cell death by inhibiting the osmotic lysis of macrophages (Weinberg et al., 2016). LDH release was significantly reduced in murine F508del macrophages pretreated with glycine after infecting them with *B. cenocepacia* for 6 h (**Supplemental Figure 3D**). Interestingly, pre-infection treatment with CFTR modulators in the presence of glycine decreased *B. cenocepacia* CFUs more efficiently than post-infection treatment did (**Figure 7E**). These results comply with previous reports that show that pre-infection autophagy stimulation enhances *B. cenocepacia* clearance in macrophages (Abdulrahman et al., 2011; Al-Khodor et al., 2014). Therefore, our data demonstrate that pretreatment of CF macrophages with CFTR modulators is more effective in enhancing *B. cenocepacia* clearance than post-infection treatment.

Because non-pathogenic *E. coli* are not being directed to the autophagosomes for their clearance (Krause et al., 2018a), we tested if the F508del macrophages were still able to efficiently clear non-pathogenic *E. coli* despite their halted autophagy capacity. Murine WT and F508del macrophages were infected with *E. coli* BL21 strain at MOI of 10:1 for 0.5, 3, and 6 h. Our results show that F508del macrophages, despite having more *E. coli* CFUs at 0.5 and 3 h, were able to control the infection at 6 h (**Figure 7F**). These data suggest that only bacteria that are destined to autophagosomes such as *B. cenocepacia* are inefficiently degraded, whereas bacteria that are not directed to autophagosomes (*E. coli*) are cleared in F508del macrophages.

### CFTR Modulators Enhance *Burkholderia cenocepacia* But Not *Escherichia coli* Delivery to Lysosomes in F508del Macrophages

**Figure 8:**
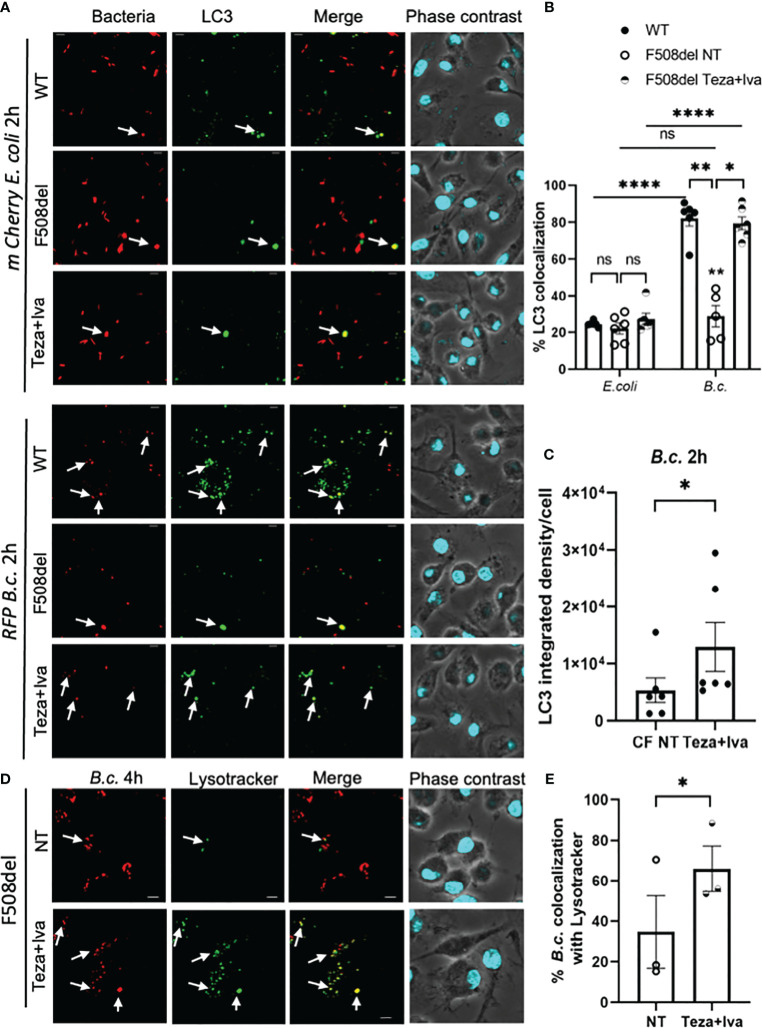
CFTR modulators enhance *Burkholderia cenocepacia* but not *Escherichia coli* delivery to autophagosomes in F508del macrophages. **(A)** LC3 immunofluorescence assay of *m-Cherry E. coli* and *RFP B. c.-*infected mouse wild-type (WT) and F508del macrophages for 2 h. F508del macrophages were either non-treated (NT) or treated with Teza+Iva for 24 h (representative n = 3 biological replicates). Scale bar = 5 µm. White arrows point to bacteria-LC3 colocalization. **(B)** % bacteria colocalized with LC3 at 2 h post-infection shown in panel **(A)** Data represent mean ± SEM calculated by scoring 5 randomly chosen fields of view with approximately 200 cells from 3 independent experiments. Statistical analysis was performed using two-way ANOVA; *p ≤ 0.05; **p ≤ 0.001; ****p ≤ 0.0001; ns, non-significant. **(C)** LC3 integrated density in F508del macrophages NT or treated with Teza+Iva for 24 h, followed by *B. c*. infection for 2 h. Data represent mean ± SEM calculated using ImageJ software 5 from randomly chosen fields of view with approximately 200 cells from 3 independent experiments. Statistical analysis was performed using Student’s paired t-test; *p ≤ 0.05. **(D)** LysoTracker Green immunofluorescence assay of *RFP B. c.-*infected F508del macrophages for 4 h. Macrophages were either NT or treated with Teza+Iva for 24 h (n = 3 biological replicates). Scale bar = 5 µm. White arrows point to *B. cenocepacia*-LysoTracker colocalization. **(E)** % *B. c.* colocalized with LysoTracker green at 4 h post-infection shown in panel **(C)** Data represent mean ± SEM calculated by scoring at least 5 randomly chosen fields of view with more than 200 cells from 3 independent experiments, colocalized RFP *B.c.* normalized to the total number of RFP *B.c*. Statistical analyses were performed using Student’s t-test; *p ≤ 0.05.

*B. cenocepacia* colocalization with autophagosomal marker LC3 at 2 h post-infection is significantly less in F508del macrophages when compared to WT ones (Abdulrahman et al., 2011; Abdulrahman et al., 2013). Since CFTR modulators enhance both autophagy and bacterial clearance in CF macrophages, we tested their effect on *B. cenocepacia* and *E. coli* trafficking within autophagosomes and lysosomes using confocal microscopy. Murine WT and F508del macrophages, either NT or treated with Teza+Iva, were infected with either *mCherry*-expressing *E. coli* or RFP-expressing *B. cenocepacia*, at MOI 10:1 for 2 h. We found no differences in the colocalization of *E. coli* with LC3 in WT, F508del NT, or Teza+Iva-treated macrophages. On the contrary, *B. cenocepacia* colocalized with LC3 in WT more significantly than in F508del macrophages. Teza+Iva treatment of F508del macrophages prior to infection increased colocalization of *B. cenocepacia* with LC3-like WT levels (**Figures 8A, B**). *E. coli* were significantly less colocalized with LC3 when compared to *B. cenocepacia* in WT cells. Additionally, measuring LC3 signal intensity in *B. cenocepacia*-infected F508del macrophages, we found that it was increased ~1.6 fold in response to Teza+Iva treatment (**Figure 8C**). Furthermore, F508del macrophages were either NT or treated with Teza+Iva and then infected with RFP-expressing *B. cenocepacia* for 4 h. Macrophages, then, were labeled with the lysosomal marker lysotracker green. Teza+Iva treatment significantly increased the delivery of *B. cenocepacia* to the lysosomes as indicated by increased colocalization of *B. cenocepacia* with lysotracker green (**Figures 8D, E**). These data demonstrate that CFTR modulators improve the degradation of *B. cenocepacia* by increasing bacterial delivery to autophagosomes and lysosomes but have no effect on bacteria that do not reside in autophagosomes.

### CFTR Modulator-Mediated *Burkholderia cenocepacia* Clearance Requires the Presence of CFTR and Effective Autophagy

While the CFTR modulators, Teza+Iva, improve autophagy and *B. cenocepacia* clearance in F508del macrophages, it is not clear if the defect in autophagy in F508del macrophages is mainly due to defective CFTR functions or due to CFTR aggregates sequestering autophagy molecules, thus impairing their function (Abdulrahman et al., 2013). To test this possibility, we used *cftr^−/−^* macrophages to examine if the complete absence of CFTR and its aggregates in macrophages impairs *B. cenocepacia* clearance. *Cftr^−/−^* macrophages were infected with *B. cenocepacia*, and bacterial clearance was compared to that in WT macrophages. We found that *cftr^−/−^* macrophages were associated with significantly higher numbers of *B. cenocepacia* CFUs at 3 and 6 h than their WT counterparts (**Supplemental Figure 4A**). Therefore, we conclude that the complete absence of CFTR expression renders macrophages more permissive to *B. cenocepacia* infection. It is unclear if modulator treatment improves autophagy, which then improves CFTR function or vice versa. Thus, we pretreated *cftr^−/−^* macrophages with the CFTR modulator combination of Teza+Iva and then infected them with *B. cenocepacia*. We found no significant differences in the number of macrophages associated with CFUs between CFTR modulator-treated and NT *cftr^−/−^* macrophages (**Supplemental Figure 4B**). Therefore, improved bacterial clearance exerted by Teza+Iva requires the presence of CFTR. Moreover, to verify if CFTR modulators can improve bacterial clearance in the absence of a functional autophagy system, we treated *atg5^−/−^* macrophages with Teza+Iva and then tested *B. cenocepacia* clearance. There was no significant improvement of *B. cenocepacia* clearance in autophagy-deficient macrophages when treated with CFTR modulators (**Supplemental Figure 4C**). These results confirm that CFTR modulators’ effect on bacterial clearance requires an effective autophagy system as well as a functional CFTR channel.

## Discussion

CF remains the most common life-shortening hereditary disease among Caucasians, with high morbidity and mortality due to chronic airway mucus obstruction, inflammation, infection, and progressive lung damage (Bruscia et al., 2009; Bruscia et al., 2011). CF has been diagnosed in many races and represents a health problem around the world (Schrijver et al., 2016). Currently, CF patients with specific mutations (i.e., G551D, F508del) can receive treatments with CFTR modulators that improve their overall wellbeing and increase their life expectancy. In fact, combinations of these modulators are being offered to F508del CF patients to target both structural and functional CFTR defects; however, their effect on fundamental macrophage functions is still unclear (Heijerman et al., 2019; Rogers et al., 2019; Ridley and Condren, 2020).

Various studies reported discordant results regarding CFTR expression, location, and involvement in bacterial clearance in immune cells such as macrophages and neutrophils (Di et al., 2006). Studies on CFTR in macrophages used blood-derived, peritoneal, alveolar, and tissue macrophages. Each of these macrophages is influenced by its tissue environment, extrinsic factors, and milieu (Bruscia and Bonfield, 2016; Turton et al., 2020). In addition, some studies that compared WT and *cftr* ^−/−^ cells did not include F508del counterparts. The complete absence of CFTR may exert different effects when compared to the low expression of mutant F508del CFTR, which aggregates and leads to unfolded protein response (UPR) (Bezzerri et al., 2019). Other studies compared cells with low expression of CFTR with cells that overexpress CFTR (Bose et al., 2019). Thus, it has been difficult to establish consensus on CFTR expression, distribution, and function in macrophages isolated from humans or mice and various tissues (blood, peritoneum, and lungs). To address these discrepancies and minimize the effect of the environment, we used naive murine BMDMs from WT, F508del, and *cftr^−/−^* mice. We also used human MDMs from healthy non-CF donors and CF^F508del/F508del^ patients to determine the location of CFTR and to confirm the findings observed in mouse macrophages.

The effect of CFTR modulators, ivacaftor and lumacaftor, on the gating and stability of mouse F508del was studied previously using an overexpression system in CHO cells (Bose et al., 2019). It was found that ivacaftor fails to improve CFTR-mediated iodide efflux in CHO cells expressing mouse F508del-CFTR. The experiment was performed at 28°C, which allows the mutant protein to reach the plasma membrane, and it was acquired from excised membrane patches. However, results from other groups indicated that mouse CFTR is more sensitive to the potentiating effect of ivacaftor than human CFTR. These results were acquired from CFTR expressed in oocytes and from CFTR in excised membrane patches (Cui and McCarty, 2015; Cui et al., 2016). Additionally, *in vivo* experiments have demonstrated that ivacaftor enhanced fluid secretion in salivary glands ducts of NOD mice that had minimal secretion prior to their treatment. Furthermore, they show that this effect was mediated *via* CFTR (Zeng et al., 2017).

Our study was performed at 37°C in the presence of both Iva and Teza. We used ivacaftor and tezacaftor at concentrations that are within the range of tezacaftor and ivacaftor plasma concentration and ivacaftor nasal tissue concentration in CF patients (Guimbellot et al., 2020; Veit et al., 2020). Under these conditions, Teza+Iva improved F508del CFTR-mediated Cl^−^ conductance at the plasma membrane, autophagy flux, and bacterial clearance in mouse F508del macrophages. It is possible that Tez+Iva combination improves the F508del CFTR channel function in the plasma membranes of primary macrophages, which then mediates intracellular effects. Yet it is conceivable that CFTR is involved in autophagy activity independently of its channel function at the plasma membrane. In addition to chloride transport, CFTR is known to transport bicarbonate in most cells that express it including airway epithelial cell, which has an important role in airway surface liquid (ASL) acidification (Quinton, 2008; Byrne et al., 2015; Kunzelmann et al, 2017). CF patients elicit increased acidity of their ASL due to impaired bicarbonate transport *via* CFTR. This high acidity impairs the antimicrobial activity of certain proteins secreted in ASL and enhances airway infections (Shah et al., 2016). CFTR corrector treatment was found to enhance the bicarbonate permeability of CFTR in cells expressing CF mutation F508del (Fiore et al., 2020). Thus, defective bicarbonate transport *via* CFTR could be a contributing factor to the defective acidity inside the lysosomes in CF macrophages.

Other locations of CFTR within immune cells have been a subject of debate. Few reports using EM showed that CFTR is expressed on vacuoles harboring inert particles, and others found it on intracellular organelles, such as lysosomes (Bradbury, 1999). In contrast, studies found negligible basal expression of CFTR in cultured primary human alveolar macrophages (Lubamba et al., 2015). While one study suggested the transient presence of CFTR around bacteria in RAW cells (Barriere et al., 2009), CFTR is reported to be located on lysosomes of mouse alveolar macrophages (Sorio et al., 2011). As described above, we used 3D reconstruction and found that CFTR is present around *B. cenocepacia* in normal non-CF and to a significantly lesser extent in CF macrophages. This is the first report of CFTR recruitment to *B. cenocepacia* containing autophagosomes. But what is the role of CFTR on autophagosomes? We and others have established that *B. cenocepacia* is cleared by autophagy in healthy human and mouse macrophages (Abdulrahman et al., 2011; Assani et al., 2014), whereas in macrophages carrying the F508del mutation, *B. cenocepacia* persists due to defective autophagy. Importantly, pretreatment of CF macrophages with CFTR modulators improved their ability to degrade *B. cenocepacia*. Nevertheless, the pathogen was not eradicated. These results are supported by recent clinical data showing that patients receiving CFTR modulators have reduced bacterial burdens at the early stages of infection, but infection is not cleared (Hisert et al., 2017). Another recent study demonstrated that macrophages derived from patients on lumacaftor and ivacaftor elicit slight improvement in their ability to clear some pathogens such as *P. aeruginosa* (Zhang et al., 2018). Autophagy plays an important role in *P. aeruginosa* clearance from lungs, and macrophages are essential for the clearance of *P. aeruginosa* during lung infection (Yuan et al., 2012). Therefore, the effects of the CFTR modulators on *B. cenocepacia* clearance could be relevant to other bacterial pathogens infecting CF patients at very high rates including *P. aeruginosa*. Moreover, CFTR modulators Teza+Iva improved lysosomal acidification and bacterial degradation *via* the autophagy pathway in CF macrophages. In contrast, Teza+Iva treatment did not affect the fate of *E. coli* in CF macrophages. A recent study used nanosensors and demonstrated that the phago-lysosomal acidity was not impaired in F508del human macrophages (Law et al., 2019). Our data show that CFTR modulators selectively improve the clearance of bacteria that inhabit autophagosomes but not others. Hence, our data demonstrate that CFTR contributes to the acidification of autophagosomes and autophago-lysosomes but not to typical endosomes that do not acquire autophagy markers. This finding is important since the number of contradictory reports regarding the role of CFTR in acidification is on the rise. Several studies reported that CFTR is required for the proper acidification and re-acidification of intracellular organelles (Barasch et al., 1991; Di et al., 2006; Teichgräber et al., 2008; Deriy et al., 2009; Liu et al., 2012). Other equally elegant reports showed that CFTR does not affect the acidification of endocytic organelles in macrophages (Haggie and Verkman, 2007; Barriere et al., 2009; Steinberg et al., 2010; Law et al., 2019). They suggested that alveolar and peritoneal macrophages from *cftr^−/−^* mice are not impaired in their ability to restrict the growth of bacteria and exert no significant alteration in acidification of their phagosomes (Di et al., 2006). Similar findings were reported in CF epithelial cells. Nevertheless, several studies that reported that acidification of intracellular organelles does not require CFTR were performed in cell lines (Root et al., 1994) or relied on CFTR_inh_-172. In addition, one study showed that cation efflux rather than Cl^−^ influx is responsible for the acidification of lysosomes (Steinberg et al., 2010). A recent study showed that CFTR acts as a transporter for bicarbonate and that treatment with correctors improves CFTR permeability to bicarbonate (Fiore et al., 2020). Additionally, we demonstrate here that enzymes that require a high acidic environment for their optimal function such as cathepsin D are less active within the less acidic compartments of F508del macrophages (Szulc-Dąbrowska et al., 2020). This defective autophago-lysosomal acidification is accompanied by impaired lysosomal degradation, which is corrected by CFTR modulator treatment. Thus, in our report, we employed several complementary approaches, and we demonstrate that CFTR is required for the acidification of autophago-lysosomes and proper degradation of their contents in primary macrophages.

The localization of CFTR on the autophagosomes that we have shown in our study poses an important question. Does CFTR contribute to the acidification of all vacuoles carrying any bacteria or mainly autophagosomes? The existence of different subsets of lysosomes has been reported (Zhang et al., 2010). These subgroups may differentially require CFTR for acidification. It is therefore possible that autophagosomes preferentially fuse with a specific subset of lysosomes that require CFTR to acidify (Yim and Mizushima, 2020). We propose here a model where CFTR mainly contributes to the acidification of autophago-lysosomes, but not to typical endosomes that do not acquire autophagy markers. This conclusion is corroborated by the fact that *E. coli*-containing vacuoles do not acquire LC3, yet the bacterium is efficiently degraded in CF macrophages. Our finding may explain the discrepancy in the studies performed by different groups. Therefore, when using particles or molecules that do not inhabit autophagosomes, CFTR is dispensable for the acidification of their containing vacuoles and their degradation. In addition, defective autophagic activity, altered lysosomal pH, and degradation abilities of autophagosomes in F508del macrophages are significantly improved with CFTR modulators. These results corroborate our findings above that CFTR is essential for the acidification of autophago-lysosomes and for the ability of macrophages to degrade pathogens targeted by autophagy. This result also shows that improved bacterial clearance in response to CFTR modulators in macrophages is mediated by CFTR.

Together, our study describes new biological location and function for CFTR during autophagy and offers a mechanism by which CFTR modulators improve bacterial clearance in CF cells.

## Data Availability Statement

The original contributions presented in the study are included in the article/**Supplementary Material**. Further inquiries can be directed to the corresponding author.

## Ethics Statement

The studies involving human participants were reviewed and approved by The Institutional Review Board of Nationwide Children’s Hospital. Written informed consent to participate in this study was provided by the participants’ legal guardian/next of kin. The animal study was reviewed and approved by The Animal Care and Use Committee (IACUC) of The Ohio State University College of Medicine.

## Author Contributions

Conceptualization: AB and AA. Investigation, conducting experiments, acquiring data, and analyzing data: AB, ME, KK, KH, FR-A, MG, EC-B, and AA. Writing—original draft: AB, EC-B, and AA. Writing—review and editing: AB, KK, ME, KH, SE, AA, KD, AH, MA, CC, TB, MG, SP-S, SS, EC-B, and AA. Project supervision and funding acquisition: EC-B and AA.

## Funding

Studies in the Amer and Cormet-Boyaka Laboratories are supported by NIAID R01 AI24121 and NHLBI R01 HL127651-01A1. AB and SE are supported by funding from the Egyptian Bureau of Education. AB and KH are supported by Cure Cystic Fibrosis Columbus (C3). KH is supported by NIH T32 Infectious Disease Institute, Ohio State University. KK is supported by a grant from the Cystic Fibrosis Foundation. AK is supported by funding from Taawon Welfare Association. FR-A and SP-S are supported by Cystic Fibrosis Foundation grant PARTID18P0 (SP-S). This work was supported in part by the Cure CF Columbus Translational Core (C3TC). C3TC is supported by the Division of Pediatric Pulmonary Medicine, the Biopathology Center Core, and the Data Collaboration Team at Nationwide Children’s Hospital. Grant support was provided by The Ohio State University Center for Clinical and Translational Science (National Center for Advancing Translational Sciences, Grant UL1TR002733) and by the Cystic Fibrosis Foundation (Research Development Program, Grant MCCOY19RO) and the C3 Immune Core.

## Conflict of Interest

The authors declare that the research was conducted in the absence of any commercial or financial relationships that could be construed as a potential conflict of interest.

## Publisher’s Note

All claims expressed in this article are solely those of the authors and do not necessarily represent those of their affiliated organizations, or those of the publisher, the editors and the reviewers. Any product that may be evaluated in this article, or claim that may be made by its manufacturer, is not guaranteed or endorsed by the publisher.
