# Supplementary material for: CFTR Modulators Restore Acidification of Autophago-Lysosomes and Bacterial Clearance in Cystic Fibrosis Macrophages
Source: Front Cell Infect Microbiol. 2022 Feb 16;12:819554. doi: 10.3389/fcimb.2022.819554 (PMC8890004; doi:10.3389/fcimb.2022.819554)
Supplement: Supplementary file 1 [file DataSheet_1.pdf]

Supplemental Figure 1

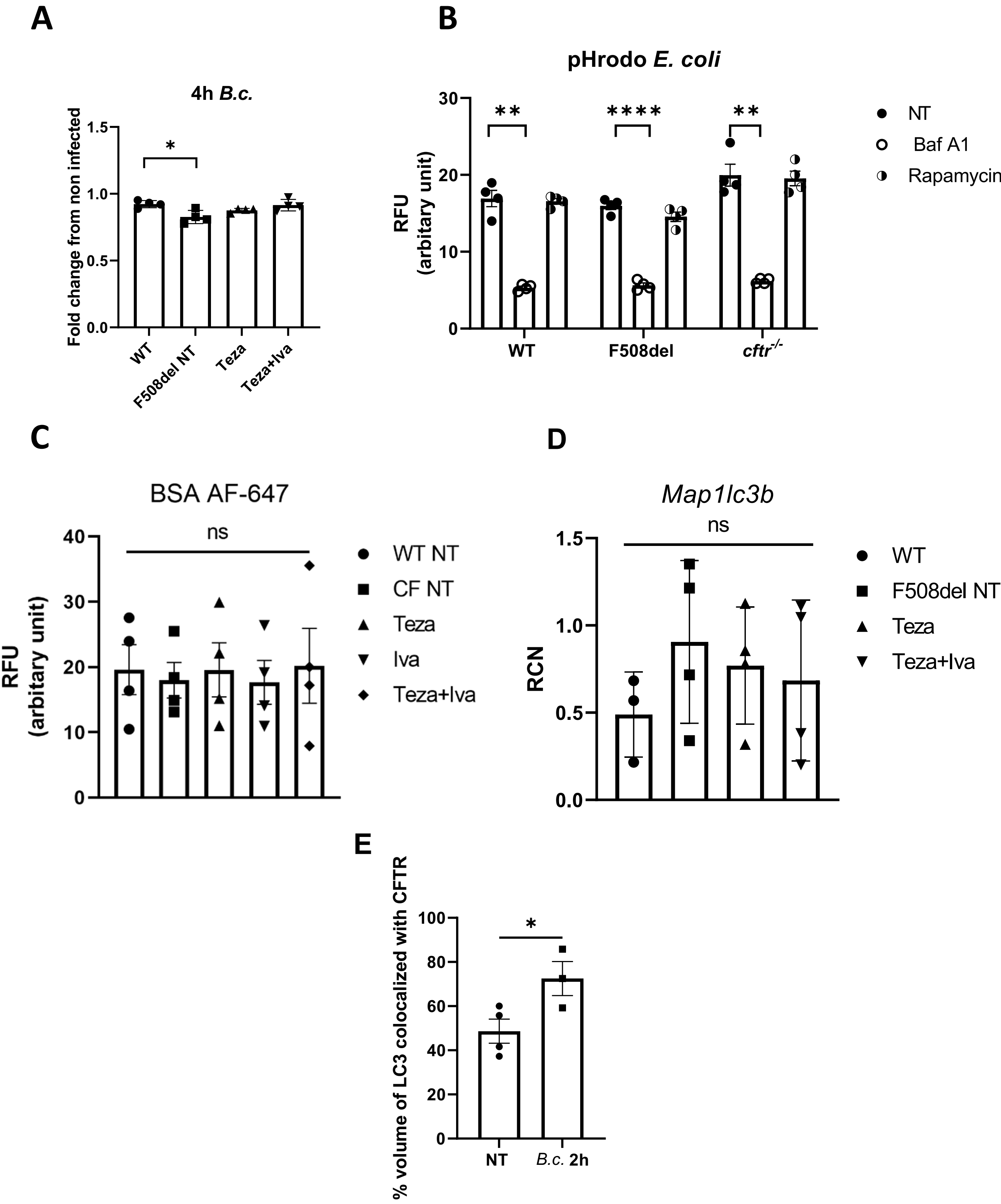

Supplemental figure 1. (A) Lysosomal acidification is impaired in F508del macrophages when infected with *B. cenocepacia*, and CFTR modulators treatment improves their acidity. WT and F508del macrophages non-treated (NT) or treated with Teza-/+Iva were infected with *B. cenocepacia* MH1K for 4 hours then stained with lysosensor green (LSG). The fluorescence of LSG was measured by a plate reader. Fold change from the non-infected cells is shown. Data represent mean ±SEM (n=4 biological replicates). *Statistical ana*lysis was performed using one-*way ANOVA*. **(B)** pHrodo *E. coli* fluorescence in bone marrow derived macrophages. WT, F508del, and *cftr*$^{-/-}$ macrophages were either non-treated, or treated with Bafilomycin A1, or Rapamycin, then incubated with pHrodo *E. coli* for 6 hours. The fluorescence intensity was measured using a plate reader and the readings were normalized to the cell number. Data represent mean ±SEM (n=1 biological and n=4 technical replicates). Statistical analysis was performed using two-way ANOVA. **(C)** BSA-AF-647 fluorescence in WT and F508del macrophages non-treated (NT) or treated as indicated on the graph. Data show mean fluorescence intensity (MFI) normalized to the total number of cells. Data represent mean ±SEM (n=4 biological replicates). Statistical analysis was performed using two-way ANOVA. **(D)** Relative copy number (RCN) of map1lc3b transcripts that are normalized to housekeeping gene gapdh. WT and F508del murine macrophages were either non-treated (NT) or treated with Teza-/+Iva for 24 hours. Data represent mean ±SEM (n=4 biological replicates). Statistical analysis was done using two-way ANOVA, ns: non-significant. **(E)** % volume of LC3 colocalized with CFTR measured in non-CF human monocyte derived macrophages that were either non infected (NT) or infected with MH1K *B. cenocepacia* for 2 hours (*B.c.* 2h). Data represent mean ±SEM calculated from 3D reconstructed images using Imaris software from at least 4 randomly chosen fields of view with an average of 30 cells per field (n=4 NT, and n=3 *B.c.* 2h). Statistical analysis was performed unpaired t-test, *, p≤0.05.

Supplemental Figure 2

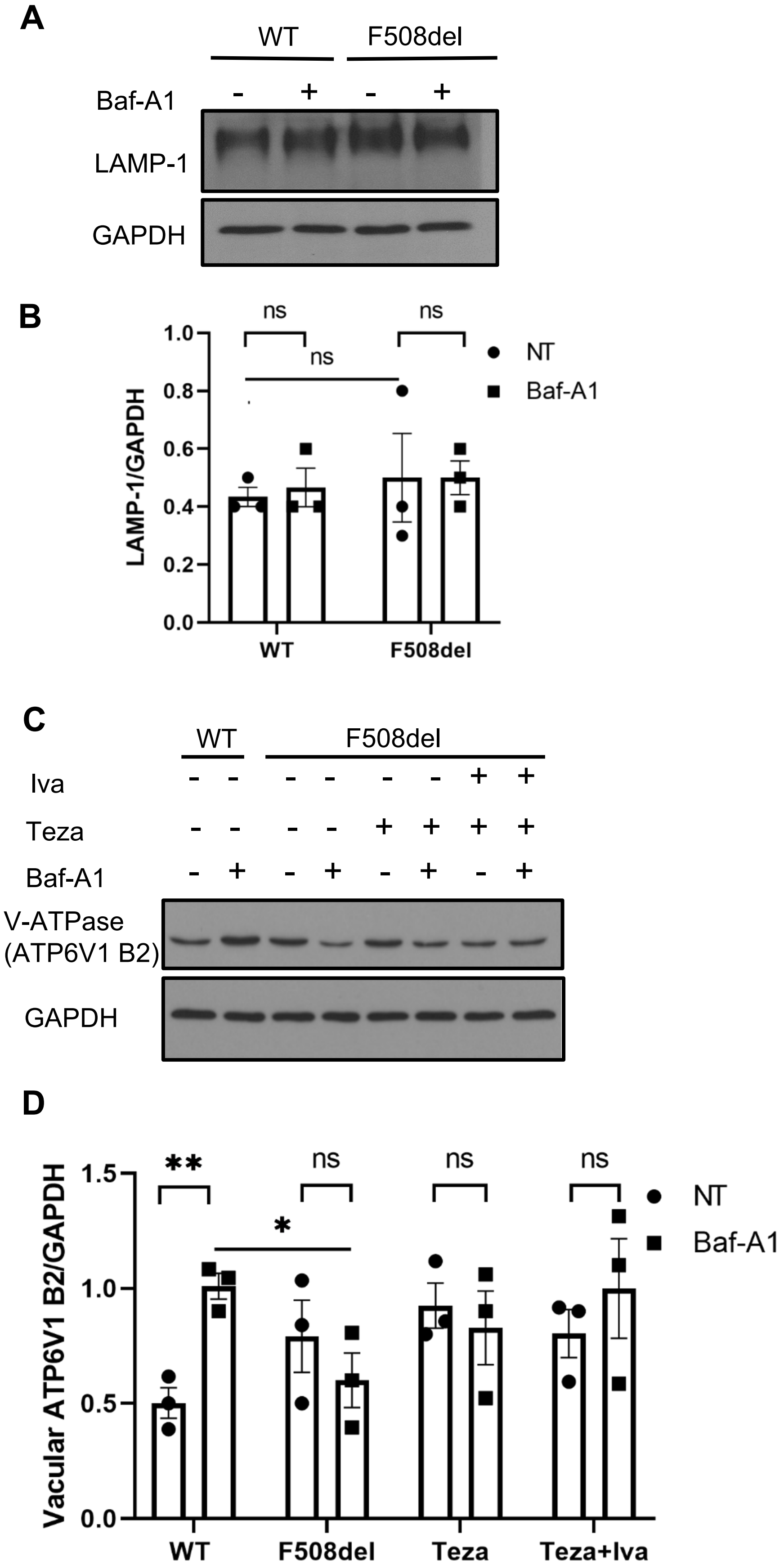

Supplemental Figure 2. The expression of lysosomal proteins is similar between WT and F508del macrophages. (A) Representative LAMP-1 immunoblot from WT and F508del macrophages either NT or treated with Baf-A1 (n=3 biological replicates). **(B)** Densitometry analysis of LAMP-1 expression in WT and F508del macrophages either NT or treated with Baf-A1. Data represent mean ±SEM (n=3 biological replicates). Statistical analysis was performed using two-way ANOVA*.* **(C)** Representative V-ATPase (ATP6V1, B2 subunit) immunoblot from WT and F508del macrophages either NT or treated with Teza -/+ or Iva for 24 hours, followed by -/+ Baf-A1 for 2 hours (n=3 biological replicates). **(D)** Densitometry analysis of V-ATPase expression in WT and F508del macrophages treated as in C. Data represent mean ±SEM (n=3 biological replicates). Statistical analysis was performed using two-way ANOVA, ***, p≤0.01.*

Supplemental Figure 3

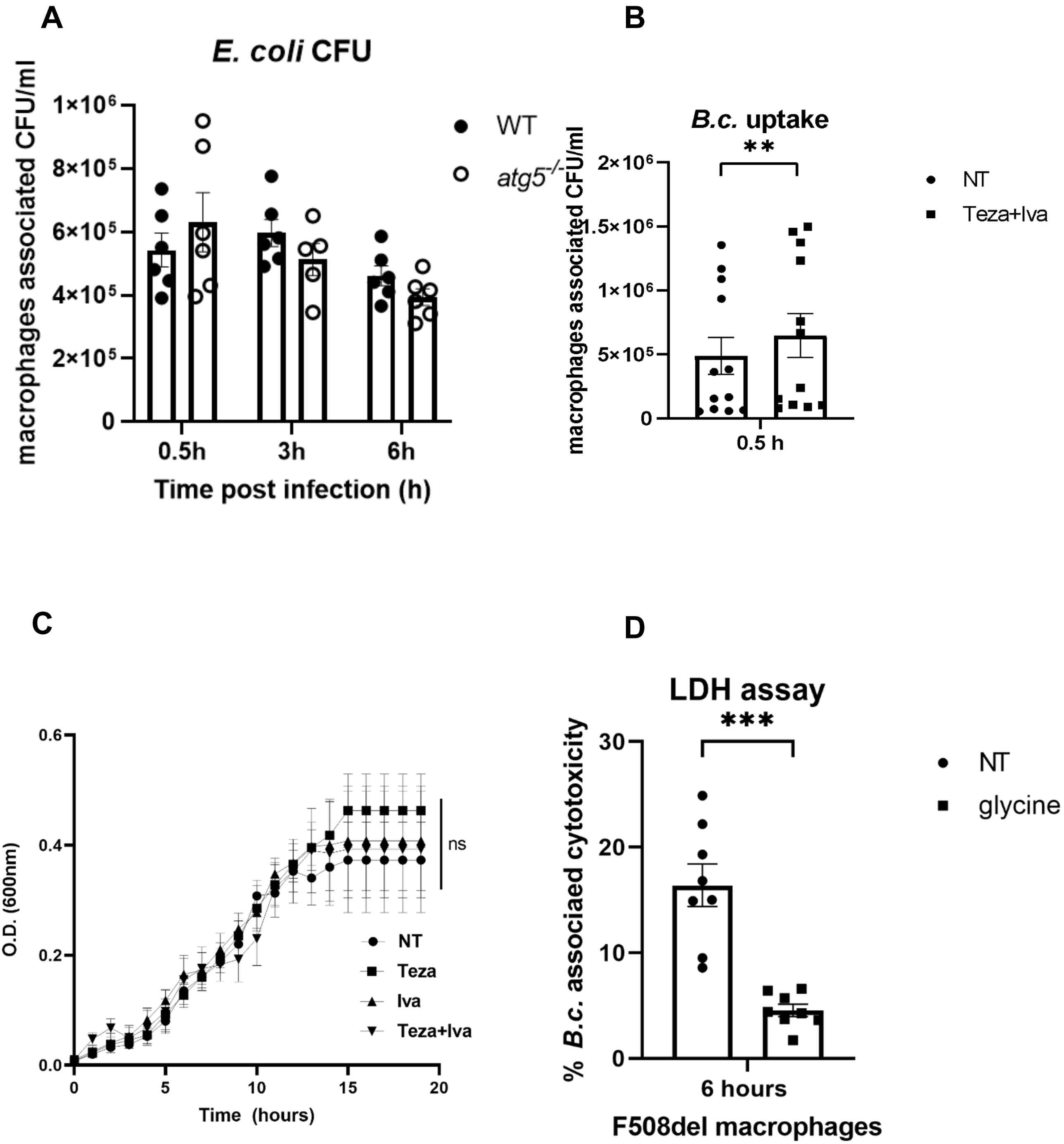

Supplemental figure 3. (A) *atg5*$^{-/-}$ macrophages efficiently clear non-pathogenic *E. coli*. WT and *atg5*$^{-/-}$ macrophages were incubated with non-pathogenic *E.coli* for 0.5, 3, and 6 hours. Colony forming units (CFUs) were quantified at each time points. Data represent mean ±SEM (n=3 biological and 6 technical replicates). Statistical analysis was performed using two-way ANOVA. **(B)** Intracellular uptake of *B. c*. at 0.5 hours of infection in F508del CFTR mouse macrophages either NT or treated with Teza +Iva (10&5µM) respectively. Data represent mean ±SEM (n=3 biological replicates). Statistical analysis was done using two-way ANOVA, ***, p≤0.01.* **(C)** *B. c*. growth in LB media either NT or in the presence of the indicated compounds. Data represent mean ±SEM (n=4 biological replicates). Statistical analysis was done using two-way ANOVA. **(D)** LDH release from *B. c.* infected mouse F508del macrophages at 6 hours post-infection. Macrophages were either NT or treated with glycine 1 hour prior to infection and throughout the course of infection. Data represent mean ±SEM (n=4 biological replicates). Statistical analysis was performed using paired t-test, ****, p≤0.001*.

Supplemental Figure 4

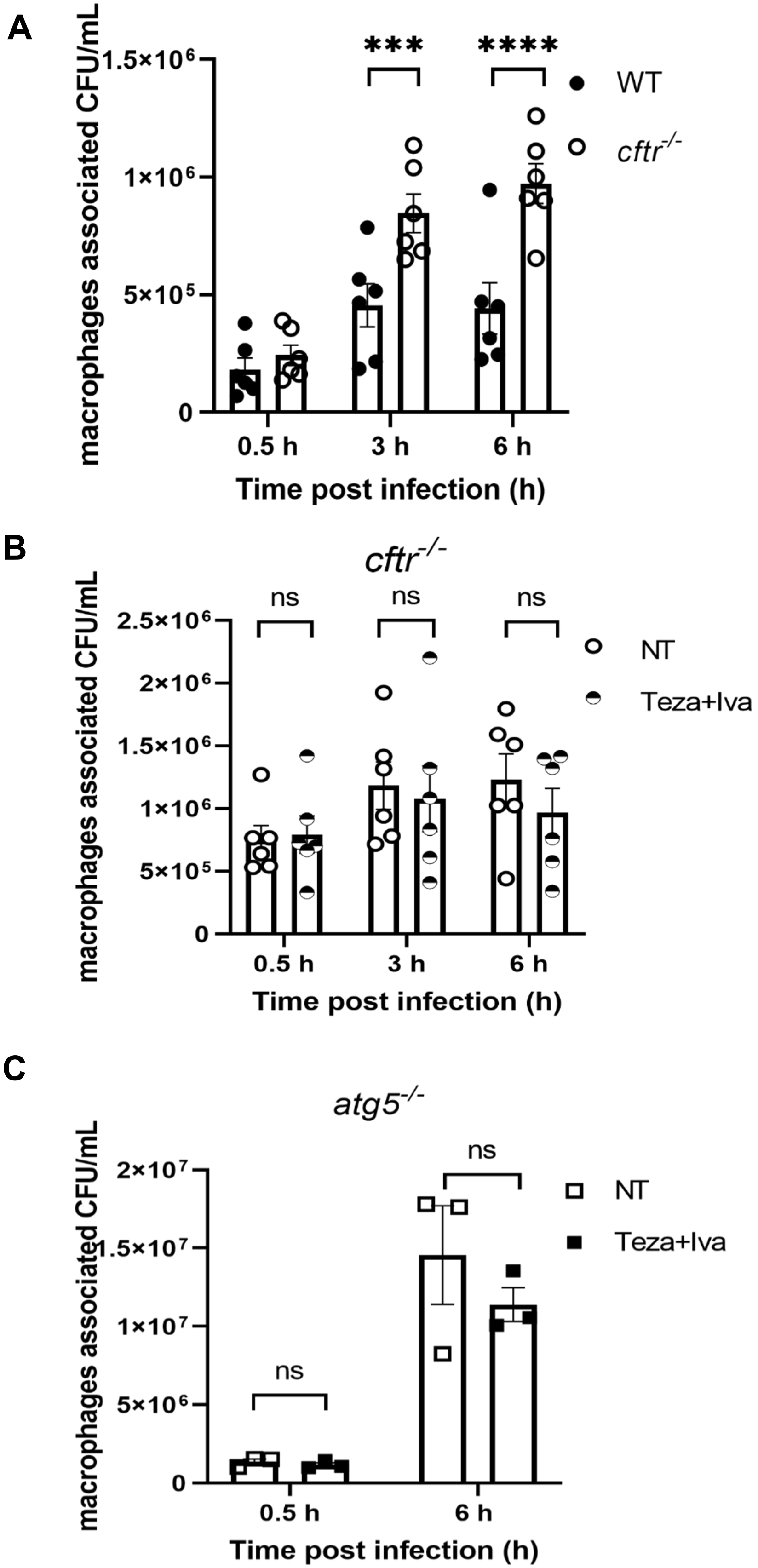

Supplemental figure 4. CFTR modulators fail to correct defective *B. cenocepacia* clearance in *cftr$^{-/-}$* and *atg5$^{-/-}$* macrophages (A) Intracellular survival of B. c. in WT and cftr-/- macrophages at 0.5, 3-, and 6-hours post-infection. Data represent mean ±SEM (n=6 biological replicates). Statistical analysis was performed using two-way ANOVA, ****, p≤0.001, ****, p≤0.0001*. **(B)** Intracellular survival of *B. c.* in *cftr$^{-/-}$* macrophages either NT or treated with Teza +Iva for 24 hours, prior to their infection, at 0.5, 3-, and 6-hours post-infection. Data represent mean ±SEM (n=6 biological replicates). Statistical analysis was performed using two-way ANOVA. **(C)** Intracellular survival of *B. c.* in *atg5$^{-/-}$* macrophages either NT or treated with Teza +Iva for 24 hours, prior to their infection, at 0.5-, and 6-hours post-infection. Data represent mean ±SEM (n=3 biological replicates). Statistical analysis was performed using two-way ANOVA, *ns, non-significant.*
